# Serum from Older Adults Increases Apoptosis and Molecular Aging Markers in Human Hippocampal Progenitor Cells

**DOI:** 10.14336/AD.2021.0409

**Published:** 2021-12-01

**Authors:** Chiara de Lucia, Tytus Murphy, Aleksandra Maruszak, Paul Wright, Timothy R Powell, Naomi Hartopp, Simone de Jong, Michael J O'Sullivan, Gerome Breen, Jack Price, Simon Lovestone, Sandrine Thuret

**Affiliations:** ^1^Department of Basic and Clinical Neuroscience, Institute of Psychiatry, Psychology & Neuroscience, King’s College London, London, UK.; ^2^Social, Genetic and Developmental Psychiatry Centre, Institute of Psychiatry, Psychology & Neuroscience, King’s College London, London, UK.; ^3^UQ Centre for Clinical Research, University of Queensland, Brisbane, Queensland, Australia.; ^4^Department of Psychiatry, University of Oxford, Oxford, UK.

**Keywords:** neural stem cells, neurogenesis, ageing, hippocampus, systemic environment, cell death

## Abstract

Age-related alteration in neural stem cell function is linked to neurodegenerative conditions and cognitive decline. In rodents, this can be reversed by exposure to a young systemic milieu and conversely, the old milieu can inhibit stem cell function in young rodents. In this study, we investigated the *in vitro* effect of the human systemic milieu on human hippocampal progenitor cells (HPCs) using human serum from early adulthood, mid-life and older age. We showed that neuroblast number following serum treatment is predictive of larger dentate gyrus, CA3, CA4 and whole hippocampus volumes and that allogeneic human serum from asymptomatic older individuals induced a two-fold increase in apoptotic cell death of HPCs compared with serum from young adults. General linear models revealed that variability in markers of proliferation and differentiation was partly attributable to use of antihypertensive medication and very mild cognitive decline among older subjects. Finally, using an endophenotype approach and whole-genome expression arrays, we showed upregulation of established and novel ageing molecular hallmarks in response to old serum. Serum from older subjects induced a wide range of cellular and molecular phenotypes, likely reflecting a lifetime of environmental exposures. Our findings support a role for the systemic enviroment in neural stem cell maintenance and are in line with others highlighting a distinction between neurobiological and chronological ageing. Finally, the herein described serum assay can be used by future studies to further analyse the effect of environmental exposures as well as to determine the role of the systemic environment in health and disease.

Several studies using heterochronic parabiosis - involving the surgical attachment of a young and old rodent so that they share a common vascular system - have demonstrated that age-related declines in stem cell function can be reversed throughout the body and central nervous system by exposure to a youthful systemic environment [1-10].

Furthermore, many of these studies have also shown that the ageing systemic environment inhibits the function of stem cells in young rodents *in vivo* and *in vitro* [2, 3, 9, 10]. One of the potential mechanisms by which the youthful milieu reverses age-related declines in stem cell function is by ‘resetting’ the molecular hallmarks of ageing to levels observed in young animals [10-13].

**Figure 1.**
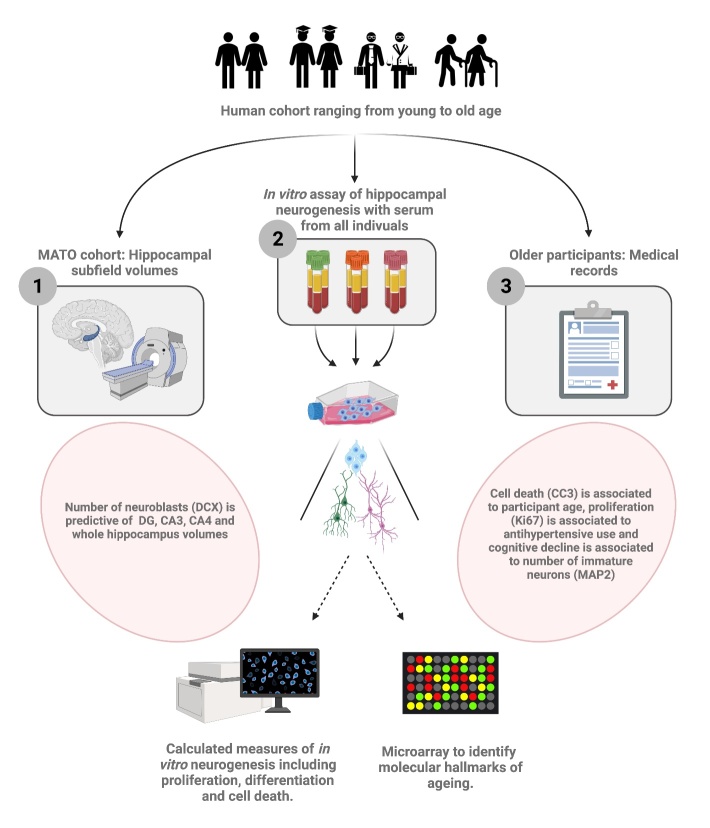
Graphical Abstract. Using two serum-donor cohorts consisting of a middle-aged to old cohort (MATO) and a cohort with young and old participants, we investigated the effect of ageing human serum on hippocampal progenitor cells. Firstly (1) using the MATO cohort, we show that participant hippocampal volumes are strongly associated to age. Second (2), using serum from both cohorts, we employ a novel *in vitro* assay to assess the effect of ageing serum on *in vitro* neurogenesis. To this end, immunostaining for markers of neurogenesis was carried out alongside a microarray analysis focused on identifying the molecular hallmarks of ageing. Interestingly, by combining results obtained in (1) and in (2) we show that neuroblast number following the application of serum is predictive of larger hippocampal subfield volumes of the corresponding serum donors. In addition (3), we use medical records of older participants to test for association between *in vivo* and *in vitro* phenotypes. We report an association between cognitive decline of older participants and Map2 positive cells. Finally, using the young and old cohort we show that age is associated to cell death following the *in vitro* assay.

With respect to neural stem cells, Villeda and colleagues demonstrated that exposure to the ageing milieu via parabiosis or injection of old rodent plasma resulted in a marked decrease in doublecortin (DCX) positive cells in the hippocampal dentate gyrus, indicating reduced neurogenesis. In contrast, exposure to the young milieu reversed age-related declines in DCX expression. These cellular changes were reflected in electro-physiological and behavioural measures whereby the ageing milieu decreased long term potentiation and impaired contextual fear memory and spatial learning in young mice that received old plasma [9]. Further to this, genome-wide microarray analysis of whole hippocampi revealed that molecular pathways related to synaptic plasticity regulation were upregulated in the aged heteroparabionts (young-old) when compared to aged isochronic parabionts (old-old) [8]. Interestingly, a recent study suggested it may be sufficient to simply dilute the age-related circulating factors to achieve the positive effects of blood heterochronicity such as increased neurogenesis [14]. The existence of adult neurogenesis, which exhibits age-related decline, is established in rodent models, but a focus of debate in humans [15-17]. This is largely attributable to the lack of robust methodologies to detect and longitudinally measure this process in human subjects [18-20]. Despite this, throughout the last decade there has been an ever-growing body of evidence supporting the notion of human adult hippocampal neurogenesis [15, 18, 21-24]. Importantly, adult neurogenesis has been repeatedly linked to age-related conditions such as Alzheimer’s disease [17, 25-28] and to environmental factors such as diet and exercise [29-31] thereby suggesting that environmental interventions aimed at potentiating adult neurogenesis could reverse ageing phenotypes and delay the onset of conditions such as Alzheimer’s Disease. Therefore, understanding how neural stem cell regulation is modulated during ageing is an important and timely goal. The effect of the environment on ageing phenotypes has also given rise to the notion that biological and chronological ageing differ and that environmental exposures, among others, are responsible for the increased heterogeneity often witnessed in older human populations [32].

In the present study (Fig. 1), we optimised a cellular assay of human hippocampal neurogenesis [33-38] to test the hypothesis that the ageing human systemic environment negatively impacts human hippocampal progenitor cells (HPCs). First, we assessed the vailidity of the model by testing for association between a known *in vivo* phenotype of ageing, hippocampal volume, and *in vitro* cellular readouts. Next, we compared the effects of human serum derived from healthy control subjects of varying age upon cellular markers of stem cell identity, proliferation, differentiation and apoptosis *in vitro*. Cellular readouts were then combined into a General Linear Model (GLM) to evaluate the contribution of epidemiological factors among older subjects. We next assessed whether molecular hallmarks of ageing were elevated in HPCs exposed to human serum derived from older persons. Finally, we employed an endophenotype approach - selecting serum samples from young and old subjects based on contrasting effects on apoptosis - to conduct an unbiased genome-wide expression array of HPCs exposed to these samples.

## MATERIALS AND METHODS

### Human hippocampal progenitor cells and culture

In this study we used the multipotent human hippocampal progenitor cell line HPC0A07/03C (kindly provided by ReNeuron Ltd., Surrey, U.K) [33]. This cell line has previously been used to investigate cellular and molecular regulation of human hippocampal neurogenesis in a controlled environment [34, 35, 38-43].

Full details of how this cell line was obtained and then immortalised by transfection with the growth promoting and telomerase upregulating gene c-Myc, that is fused to a modified oestrogen receptor are detailed herein [43, 44]. This receptor is selectively activated by the synthetic drug 4-hydroxy-tamoxifen (4-OHT) at 100 nM and c-Myc activity is therefore strictly dependent on the presence of 4-OHT (Supplementary Methods).

HPC03A/07 cells were grown in reduced modified media (RMM) consisting of Dulbecco’s Modified Eagle’s Media/F12 (D6421, DMEM:F12, Invitrogen, UK) supplemented with 0.03% human albumin (Albunorm 20%, Octapharma, UK), 100 µg/ml human apo-transferrin (T1147, Sigma), 16.2 µg/ml human putrescine DiHCl (P5780, Sigma), 5 µg/ml human recombinant insulin (I9278, Sigma), 60ng/ml progesterone (P8783, Sigma), 2 mM L-glutamine (G7513, Sigma) and 40 ng/ml sodium selenite (S9133, Sigma). To maintain proliferation, 10 ng/ml human bFGF (EC100-18B, Peprotech), 20 ng/ml human EGF (AF100-15-500, Peprotech) and 100 nM 4-OHT (H7904, Sigma) were added. The cell culture media is free of any serum unless stated as a treatment in the experimental conditions. In addition, when serum is added, we also further supplement our media with 0.5 mg/ml Penicillin Streptomycin (10,000 U/ml, 15140-122, Life Technologies).

HPCs were routinely passaged using acutase (A1110501, Sigma) and cultured on tissue culture flasks (Nunclon, Denmark) coated freshly with 20µg/ml mouse laminin (L2020, Sigma) at 37^o^C, in saturated humidity and 5% CO_2_. For optimal cell growth, medium was changed fully every second day. During normal expansion, HPC03A/03C cells proliferate with a doubling time of 48-72 hours (70-80% confluence) [33].

**Figure 2.**
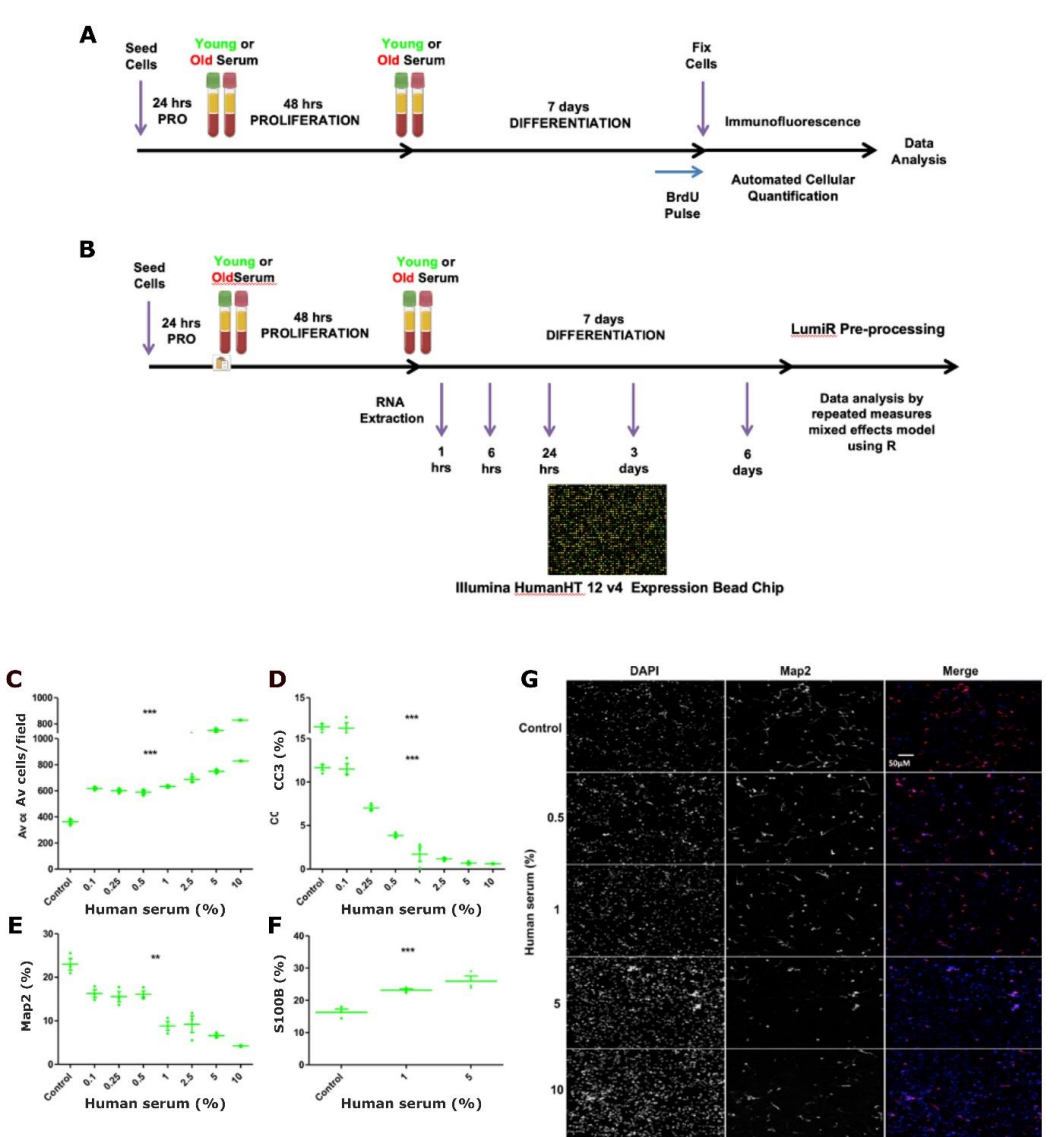
Serum assay timeline and optimal serum concentration. (A) Optimised assay timeline to evaluate the impacts of the human systemic environment on human. (B) Experimental timeline for qPCR and whole-genome expression analysis by microarray. (C-G) Human serum concentration dose-response curves for the differentiation phase of the assay. Culturing human hippocampal progenitors with increasing concentrations of serum (as a percentage on the x axis) increased (C) cell number, reduced (D) apoptotic cell death (CC3 as % of total cells) (E) immature neurons as measured (Map2 as % of total cells), and increased (F) astrogliogenesis (S100β as % of total cells). (G) Representative images of a dose-dependent decrease in Map2 staining (red fluorescence), also highlighting the increase in cell number through DAPI staining of cell nuclei. n = 3, using serum from a young male (aged 23 years). one-way ANOVA conducted across all concentrations (post-hoc comparisons not presented), ** P < 0.001, *** P < 0.001. Error bars = SEM. Scale bar applicable to all images.

### Serum assay: Proliferation and Differentiation assay phases

HPCs were plated on 96-well plates (Nunclon, Denmark) at a density of 1.2×10^4^cells per well and cultured in the presence of EGF, bFGF and 4-OHT and treated with human serum during the initial proliferation phase for 48 hours and in the subsequent differentiation phase for 7 days (total treatment of 9 days Fig. 2A). 5′-bromodeoxyuridine 10μm (BrdU, B5002, Sigma) was added to the culture media 4hrs prior to the end of the incubation to label proliferating cells. Following incubation cells were fixed with 4% paraformaldehyde in preparation for immunostaining.

### Recruitment of middle-aged to old (MATO) cohort

The MATO cohort from which serum samples were derived was recruited by the O’Sullivan group. Participants used in this study were cognitively healthy control individuals recruited as part of the larger STRATEGIC study investigating memory performance in stroke patients [45]. The exclusion criteria included moderate to severe head injury, diagnosis of a cognitive, neurological or major psychiatric disorder, self-referral for cognitive symptoms and a mother tongue other than English. As Magnetic Resonance Imaging (MRI) was used, all its pertaining exclusion criteria, such as presence of pacemakers and penetrating eye injury, were applied as well. Serum samples of 25 individuals (5 females, age range 52.6 - 89.4 years of age, mean age 73.2 ± 9.5 years) were available, of these 19 participants (4 females, age range of 55.6 - 89.4, mean age 74.5 ±8.9), also had available cognitive score data. Serum samples from this cohort were a unique resource which has been depleted by this study.

### Imaging in MATO cohort

MRI were carried out using a 3T General Electric MR750 scanner at the Clinical Research Facility at King’s College Hospital.T1-weighted images were acquired to obtain 192 sagittal slices each with a thickness of 1.2mm, a field of view of 270mm, a 1mm in-plane resolution and an acquisition matrix of 256X256. FreeSurfer (Version 6.0) (Iglesias et al., 2015; Pereira et al., 2014) was used to analyse volumetric T1-weigthed images. This software uses labels derived from an *ex-vivo* atlas of hippocampal subfields to segment the hippocampus in the acquired images. It implements an algorithm that uses a generative model and Bayesian inference. The *ex-vivo* atlas was derived from post-mortem images which were labelled manually. In this study, the resulting hippocampal segmentations were checked visually for concordance with hippocampal boundaries by a single rater. According to these segmentations, volumes for each subfield were measured and normalised to intracranial volume extracted from FreeSurfer. This created a set of normalised subfield volumes per hemisphere per person, the volumes of each hemisphere were summed to obtain the values used in the analysis. Of the resulting subfields the CA1, CA3, CA4, dentate gyrus and the subiculum were used in the analysis as well as whole hippocampal volume.

### Recruitment of young and old healthy control subjects

The young cohort from which serum samples were derived were recruited through internal advertisements throughout the Institute of Psychiatry, Psychology and Neuroscience between the period of November 2012 and June 2014. Serum samples from 30 young individuals (15 females, mean age of 27.1 ± 5.03 years) and 35 old individuals (15 females, mean of 77.6 ± 6.43 years) were collected. Serum was collected at one time-point only, except for one study where two young participants donated blood in the morning and afternoon, repeating this on the following day and one week later. All participants were required to confirm their good health and provide consent by means of a questionnaire as part of the Alzheimer’s disease biomarker programmes outlined below.

Serum samples derived from old control subjects, deemed cognitively healthy by extensive clinical testing were obtained from two ongoing Alzheimer’s disease blood biomarker discovery programmes: 1) The London cohort from AddNeuroMed, a multicentre European study [46] and 2) The Maudsley Biomedical Research Centre Dementia Case Registry at King’s Health Partners - all samples were collected in London using identical protocols. On the Mini Mental State Examination (MMSE), for example, subjects scored greater than 27 out of 30 points indicating normal condition, and results were adjusted for age and educational attainment. Exclusion criteria included other neurological or psychiatric disease, significant unstable systemic illness or organ failure and alcohol or substance misuse. Serum samples from this cohort were a unique resource which has been depleted by this study.

### Processing of serum samples

All subjects were required to fast for two hours before blood sample collection; only water or fluids containing no milk or sugar were allowed during the fasting period. Serum and plasma were first fractionated from blood cells and stored at -80^o^C. Samples were then aliquoted into appropriate volumes and again stored at -80^o^C for future use (i.e., each sample used went through one freeze thaw cycle). At the point of use, samples were thawed at 37^o^C with periodic swirling. Once completely thawed, appropriate dilutions of serum were swiftly added to cell culture medium prior to incubation with HPCs.

### Immunofluorescence

PFA-fixed cells pulsed with BrdU were first incubated with 2N hydrochloric acid for 25min at room temperature followed by neutralisation with 0.1M borate buffer for 10 min and x2 washes with phosphate-buffered saline (PBS). Next, 50µl of blocking solution comprising 5% normal goat serum (g9023, Sigma) in PBS containing 0.3% Triton-X (93443, Sigma) were added to all wells for 60min at room temperature. Primary antibodies to measure proliferation, differentiation and cell death (Supplementary Table 5) were diluted in appropriate concentrations of blocking solution buffer at 30µl/well and left at 4°C overnight. The next morning, cells were washed, incubated in blocking solution for 30min and then incubated with appropriate fluorescently tagged secondary antibodies (Alexa 488 goat anti-rat, 1:500, A21208; Alexa 555 goat anti-rabbit, 1:500, A31572; Alexa 488 goat anti-rabbit, 1:500, A21206; 555 goat anti-mouse, 1:500, A31570 - all from Life Technologies) at 30µl/well for 2h at room temperature. After 3 washes, cells were counter-stained with 300µm DAPI at 50µl/well for 5 minutes and washed on 3 more occasions. Automated quantification of immunofluorescence is detailed in the supplementary materials.

### Epidemiological factors

Whilst cognitively healthy and free from neuropsychiatric conditions, many of the old persons had clinical histories and ongoing issues with rheumatic, metabolic, cardiovascular and inflammatory events as listed in Supplementary Table 1. Moreover, many of these conditions necessitate treatment with a multitude of different pharmacological agents. These factors were divided into appropriate groups: “Inflammatory” (encompassing inflammatory conditions like arthritis and diabetes) and “Anti-hypertensives” (subject currently taking anti-hypertensive agents). Other metabolic and cardiovascular parameters such as waist circumference and blood pressure are also included in Suplementary Table 1. Finally, assessments of cognitive function (Mini Mental State Examination - MMSE, Clinical Dementia Rating - CDR, Consortium to Establish a Registry for Alzheimer’s Disease - CERAD scale), depression (Geriatric Depression Scale) and deterioration (Global Deterioration Scale) are also included in the table and analyses. Across the cohort, missing data points for each of the variables was prevalent.

### A backward elimination stepwise regression and General linear models

Using R (www.r-project.org/), co-variates (Age, Education, Gender, Deterioration, Depression, Hypertension, Inflammatory, Statins - Supplementary Table 1), and cellular readouts (Average Cell Number, Ki67, CC3, DCX, Map2) were combined into a General Linear Model (GLM) that allows analysis with non-normal errors and non-constant variance. Applying GLM to these datasets is a multiplicative approach, whereas linear analysis is additive. Cognitive scores, waist and blood pressure were excluded from the model owing to missing data points for many subjects or there being very low variation among the old cohort on these metrics (e.g., MMSE).

A backward elimination stepwise regression was also conducted, which starts with all the above variables and successively removes each variable contributing least to the model based on the lowest Akaike information criterion (AIC) - a relative estimate of the information lost (i.e., the quality of the model) when a given model is used to represent the process that generates data. When each of the steps has taken place, the results indicate co-variates that are explaining significant proportions of variance and thus ought to be included or accounted for in subsequent assembly of a GLM and future analyses. Refined models were then tested by an analysis of deviance to test their goodness of fit.

### Quantitative real-time Polymerase Chain Reaction (qPCR)

Methods used for total RNA extraction and quantification of concentration and purity; reverse transcription and amplification primer design and testing of PCR specificity are detailed in the supplementary materials.

### Housekeeping gene selection (candidate genes and microarray validation)

Vimetin (VIM), ribosomal protein large P2 (RPLP2) and gamma-actin (ACTG1) (Supplementary Table 7) were identified as suitable housekeepers based on microarray data, as they showed the least variability when the standard deviation of expression is divided by mean (also known as coefficient of variation) expression across all conditions (young and old serum) and time-points (1, 6, 24, 72 and 144 hrs) [38].

### Sybr green qPCR

Experiments were carried out following the sample maximisation approach, where all samples assayed for the same target are included in the same plate [47]. White welled qPCR plates (AB-0900/W, ThermoFisher) were exposed to UV for 10-20 minutes in the UV Stratalinker 1800 (Stratagene) to crosslink DNA prior to plating, to damage possible contaminant DNA and prevents its amplification during polymerase mediated elongation. The reaction comprised of template nucleic acid (25 or 33 ng of cDNA or 10 μl of the 1 in 30 or 40 diluted cDNA), 4µl 5X HOT FIREPol® EvaGreen® qPCR Mix Plus ROX (08-24-00008, Solis BioDyne, Estonia), 0.2 μM primer mix, and nuclease-free H_2_0 to make a total reaction volume of 20 μl. All PCR reactions were carried out with two negative controls for each primer pair, where the template nucleic acid was replaced with H_2_0. qPCR reactions were carried out on a Chromo4™ Real-Time PCR detector (Bio-Rad). using the following parameters: Initial denaturation (95ºC for 15 minutes), followed by 45 cycles of denaturation (95ºC for 30 seconds), annealing (60ºC for 30 seconds) and extension (72ºC for 30 seconds). Fluorescence was recorded at the end of each cycle. A melt curve was carried out from 60ºC-95ºC with a 10 second hold and a plate read at every 1ºC increment and visually assessed. Reactions were carried out in technical duplicate. The Pfaffl mathematical model for relative transcript quantification was used for data analysis [48].

### MitoTracker and image analysis

HPCs were incubated with MitoTracker® (M22425, Life Technologies) for labelling of active mitochondria as per the manufacture’s instructions. Cells were imaged and analysed using the Cell Insight imaging platform, in addition, thresholds for positive staining were also determined by quantifying the ‘% high’ population, defined as the proportion of cells with average intensity values greater than one standard deviation from the mean, this proportion of cells contained the largest number of active mitochondria.

### Western blotting

Methods for: lysate collection, determining protein concentration; preparing and loading samples, running gels and transferring to nitrocellulose membrane and staining membrane and antibodies used are detailed in supplementary materials. Immunofluorescence was quantified using the Odyssey system and software (Li-Cor). Images for channel 700 (680 wavelength of 2^o^ antibody) and channel 800 (790 wavelength of 2^o^ antibody) were acquired at intensity values between 6 and 9. Images were analysed by the background method based on median values. Rectangles were positioned over corresponding bands and raw data values for fluorescence were exported. Values for the phosphorylated form of the protein were normalised to values for total form of the protein and analysed as relative changes.

### Endophenotype approach

The endophenotype approach was based on the contrasting effects of serum samples on apoptotic cell death of HPCs. To this end, three young subjects with the lowest CC3 levels and three old subjects with highest CC3 levels were selected to investigate the mechanisms responsible for divergent cell phenotypes. These serum samples were re-run in the cellular assay of hippocampal neurogenesis and RNA was harvested at 5 time-points during differentiation at 1, 6, 24, 72 and 144 hours (data not shown), to evaluate early and late-gene expression changes. Comparing phenotypes at opposite ends of a biological measure(s) - in this case, serum induced levels of apoptosis - has the potential to reveal molecular mechanisms related to different subsets of a cohort and overcome age-linked variability at the group level for older subjects.

Samples were then run on a whole genome expression array (see details below). The filtered and processed microarray data were analysed using a mixed model with a repeated measures design in which the response (gene expression) is continuous and measured at fixed time points (1, 6, 24, 72 and 144 hours) and for the fixed effect of age (young vs. old). 141 probes reached a significance threshold *P* value of < 0.01 (uncorrected for multiple comparisons) for differential expression at one or more of the 5 time-points (1, 6, 24, 72 and 144 hours) following exposure to young or old serum (see Supplementary Table 4 for the full list).

### Microarray

#### Preparation and quantification of samples

RNA integrity numbers (RINs) were assessed using the Agilent Bioanalyzer (G2938-90034, Agilent Technologies, UK) according to the manufacture’s instructions. All samples had RINs of greater than 9, indicating good quality and pure RNA. RNA concentrations were quantified using the Quant-iT™ RiboGreen® RNA reagent kit (R11490, Thermo Scientific) following the manufactures instructions. 200 ng of sample RNA was prepared in 11µl nuclease-free H_2_0 in preparation for labelling and amplification using the TotalPrep™-96 RNA Amplification Kit (4393543, Illumina, US), following the manufacturer’s instructions. The concentration of labelled RNA was checked again and 750 ng was loaded onto bead chips for hybridisation. Labelled RNA samples were processed on Illumina Human HT-12 v4 Expression BeadChip (Illumina Inc., USA) according to manufacturer’s protocol.

#### Processing of data in genome studio and in LumiR

The Lumi (Bioconductor) package in R was used for quality control, background correction, variance stabilization, quantile-normalization, log-transformation and gene annotation [49]. Genes were then filtered based on detection values generated by Genome Studio. Expression probes had to reach the detection p-value threshold <0.01 (filter 1) and had to be detected in at least two of three samples in either the young or old group for at least one of the five time-points (filter 2), and if not, they were excluded.

#### Repeated measures mixed effects model and analysis

The specialised mixed model has the response of gene expression as continuous and is measured at fixed time points (1, 6, 24, 72 and 144 hours). The model includes both within subject and between subject effects as well as fixed effects (also known as factors) which refers to variables for which levels in the study represent all levels of interest (young *vs.* old). Other variables for which the model is to be adjusted (e.g. gender, batch effects) are also be included in the model as fixed factors. We also included “ε”, an “error term” to represent the deviations from our predictions due to factors that we cannot control experimentally, attributable to variation between humans and the complex nature of serum. In this model, an interaction term for time points and young vs. old is included. This model enables the expression levels of each probe to be related to each of the factors and the interaction term. Each probe was ascribed a *P*-values in relation to each of the factors. Corrections for multiple comparisons were achieved through the False Discovery Rate.

### Pathway analysis

#### Ingenuity pathway analysis

Probes with a *P*-value < 0.01 in the young vs. old analysis were also analysed for functional molecular networks using ingenuity pathway analysis (IPA, http://www.ingenuity.com, Qiagen, USA). Networks are assembled based on gene/molecule connectivity with other gene/molecules. A key assumption to this approach is the more connected a gene/molecule, the more influence it has and the more “important” it is. The significant probes were also analysed in terms of diseases and disorders, molecular and cellular functions and physiological system and development. Networks and pathways with a P-value < 0.05 were regarded as significant.

### Statistical analyses

For analysis pertaining to the MATO cohort, Spearman correlations were carried out using SPSS and GraphPad Prism software. If a significant correlation was found linear regressions were also carried out. Age regressed values were used for regressions on cell readouts and hippocampal volumes Bonferroni correction was applied to correct for multiple testing. Statistical analyses for immunofluorescence count, qPCR and Western blot relative expression data were performed with Prism 6 (GraphPad Software, USA). The Kolmogorov-Smirnov test was used to test data sets for normality. Comparisons between different concentrations of serum were carried out using appropriate parametric or non-parametric one-way ANOVA with post hoc comparisons performed using the Bonferroni correction method. Comparisons of cellular read-outs and relative expression (gene and protein) in response to young *versus* old serum were conducted at the group level using appropriate parametric or non-parametric t-tests pending testing of assumptions of a normal distribution.

## RESULTS

### In vitro cellular readouts are associated to hippocampal volumes of serum donors

**Figure 3.**
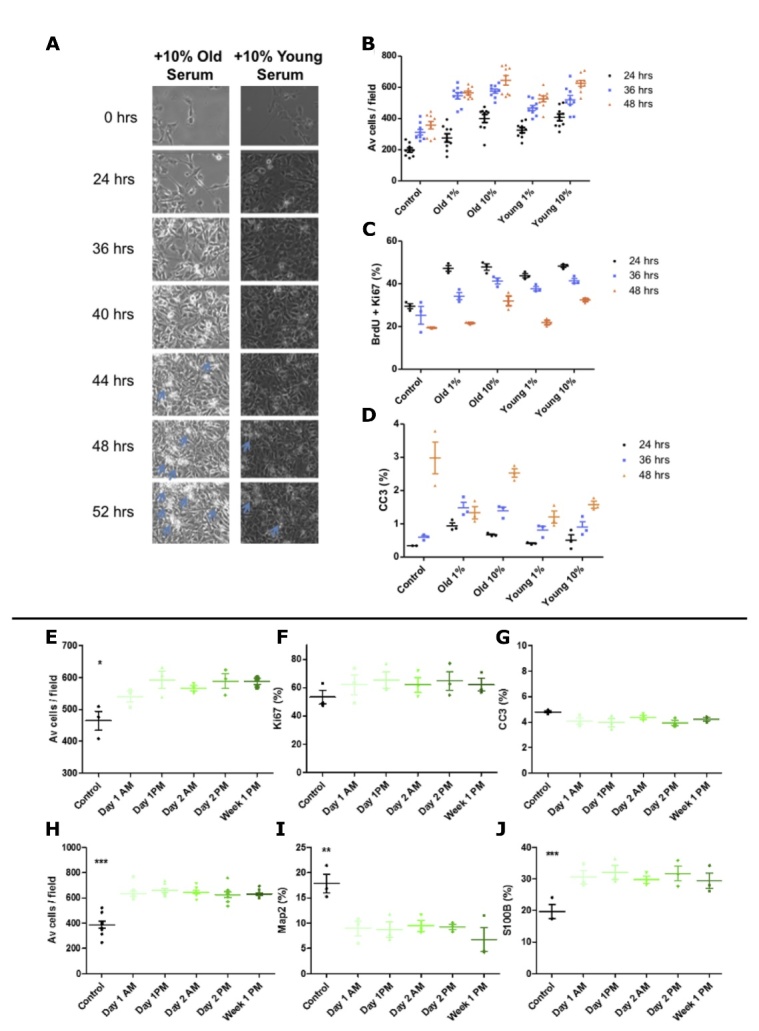
Serum assay optimisation. (A-D) Young (age = 23) male *versus* old (age = 85) male serum, live cell imaging and corresponding quantification at 24, 36 and 48hrs. (A) Live cell imaging - blue arrows indicate both increased cellular debris resulting from death with increase culture length. (B) Average number of hippocampal progenitor cells per field increases with time and appears to plateau at 48 hours. (C) Co-labelling for BrdU and Ki67 reveals progressive decrease in proliferation up to 48 hours. (D) Staining for the apoptotic marker CC3 reveals significant increases in cell death with time and that is proportionally decreased with an increase in serum. B = total number of cells divided by 15 fields, C and D presented as % of total number of cells. 15 fields were analysed per well, n = 3 technical replicates; for Av cells / field n=6 technical replicates, statistics not shown, error bars = SEM. (E-J) Time of serum donation does not alter expression levels of markers of hippocampal neurogenesis *in vitro.* Hippocampal progenitors were cultured with 1% human serum collected at multiple time-points (x-axis) from a young male (aged 23yrs) to obtain read-outs during the proliferation phase of the assay (A) cell number, (B) proliferation (Ki67 as % of total cells) (C) apoptotic cell death (CC3 as % of total cells) and differentiation phase of the assay (D) cell number, (E) immature neurons (Map2 as % of total cells) (F) astrocytes (S100β as % of total cells). n =3, one-way ANOVA conducted across all conditions (post-hoc comparisons not presented) reveals significant effect of serum compared to serum free control conditions, * P < 0.05, ** P < 0.001, *** P < 0.001. Error bars = SEM.

Given the established potent effects of serum on cellular proliferation, viability and fate [50], the serum assay was first optimised to determine the optimal concentration of human serum (Fig. 2C-G) and assay length (Fig. 3A-D) to be used for subsequent experiments as well as the effect of intra-individual serum variability over time (Fig. 3E-J). Post-optimisation, HPCs were cultured with a concentration of 1% serum obtained from participants from the Middle Aged To Old (MATO) cohort (aged 52 to 89) and the young (average age 27) and old (average age 77) cohorts, as part of this newly established *in vitro* serum assay outlined in the methods section (Fig. 2A).

First, we used the MATO cohort as a proof of concept to assess whether *in vitro* readouts following the serum assay relate to *in vivo* phenotypes of the serum donors. To this end, we investigated whether cellular readouts were also associated with serum donor age. Immunocytochemical analysis followed by Spearman’s correlations revealed no association between the expression of cellular markers or cells number following the serum assay and serum-donor age alone (Supplementary Fig. 1).

**Figure 4.**
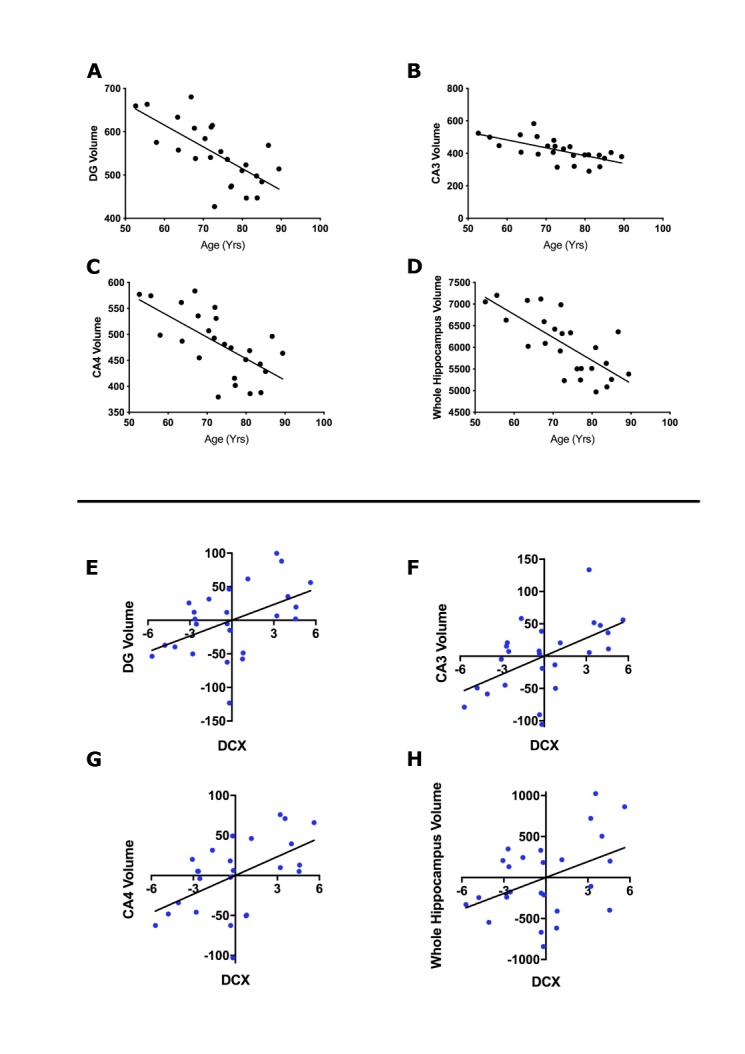
Effect of age on hippocampal subfield volume. Scatterplots showing linear regressions assessing the relationship between participant’s age in years and hippocampal subfields (A) Dentate Gyrus (DG) (Beta= -5.02, SE=1.10, p= 0.0001*, r^2^= 0.47), (B) CA3 (Beta= -4.83, SE= 1.16 , p= 0.0004*, r^2^= 0.43), (C) CA4 (Beta= -4.14, SE= 1.00, p= 0.0004*, r^2^= 0.43), (D) Whole hippocampus (Beta= -53.15, SE= 10.29, p= <0.0001*, r^2^= 0.54). Linear regression line is shown on each graph. Values following linear regressions are reported. Bonferroni correction was used to account for the 6 multiple comparisons, the significance threshold for this analysis was therefore set to 0.0083. * denotes results surviving multiple testing correction. Associations of neuroblast marker to hippocampal subfield volumes. Scatterplots showing the association between neuroblasts (DCX expression) following serum incubation and hippocampal volumes. In each graph, the x-axis shows the age-regressed percentage of HPC0A07/03A cells positive for DCX expression and the y-axis shows the age-regressed volume of the specific hippocampal subfields (E) Dentate Gyrus (DG) (Beta= 7.82, SE= 3.00, p= 0.016, r^2^= 0.23), (F) CA3 (Beta= 9.31, SE= 3.00, p= 0.005, r^2^= 0.30), G) CA4 (Beta= 7.80, SE= 2.63, p= 0.007, r^2^= 0.28), (H) Whole hippocampus (Beta= 64.68, SE= 28.80, p= 0.035, r^2^= 0.18). Linear regression line shown. Values following linear regressions are reported. Bonferroni correction was used to account for the 36 multiple comparisons (6 subfields and 6 markers), the significance threshold for this analysis was therefore set to 0.0014. No association survived multiple testing correction.

Next, we tested the association of cellular readouts following the serum assay to serum donor’s hippocampal subfield volumes. Each relevant subfield was first tested for association to participant’s age. This revealed strong associations between each subfield tested and serum-donor age. Older age was found to be predictive of decreased subiculum, dentate gyrus, CA1, CA3, CA4 and whole hippocampus volumes (Fig. 4A-D and Supplementary Fig. 1). Once the association of subfields and hippocampus volume with age was confirmed, cellular markers were tested for association to each subfield’s volume and to whole hippocampus volume. Markers for apoptosis (CC3), DNA damage (H2AX), immature neurons (Map2), neuroblasts (DCX) and proliferation as well as cell number were employed. The percentage of positive cells for each marker following incubation with serum was tested for association to the serum donor’s subfield and whole hippocampus volumes. Values were age-regressed to account for the strong effect of age on hippocampal volumes. No association was found between volumes and CC3, Map2, Ki67 expression or cell density. However, the number of neuroblasts, as shown by DCX expression in the HPCs, correlated with dentate gyrus, CA3, CA4 and whole hippocampus volume (Fig. 4A-D). In addition, the level of DNA damage, shown by H2AX expression in the HPCs, correlated with subiculum, CA3, CA4 and whole hippocampus volumes (Supplementary Fig. 2). These associations did not survive Bonferroni multiple testing corrections.

### Older adult serum increases apoptosis of human hippocampal progenitor cells

**Figure 5.**
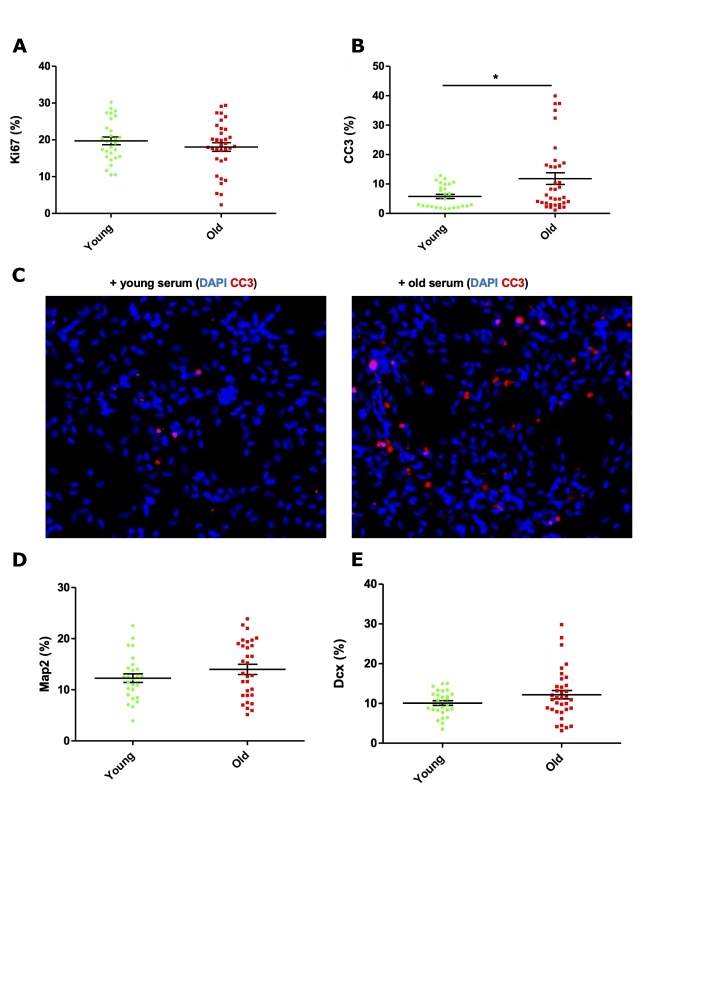
Effect of young and old human serum on proliferation, apoptosis and differentiation of human hippocampal progenitor cells. (A) Ki67 - proliferating cells; (B and C) Cleaved Caspase 3 (CC3) - apoptotic cell death; (D) DCX - neuroblasts; (E) Map2 - immature neurons following 9 days of culture with young (n=30, 15 females, mean age of 27.1 years) or old (n=35, 20 females, mean age of 77.6 years) human serum at a concentration of 1%, 3 technical replicates per sample. All presented as % of total number of cells. Unpaired student and Mann Whitney t-tests used as appropriate. * P < 0.05. Error bars = SEM.

Given evidence of association between *in vitro* readouts and *in vivo* phenotypes in the MATO cohort but the lack of association between *in vitro* readouts and serum donor age, the effect of the systemic environment was investigated further using a cohort with two distinct young and old groups. After 9 days of culture, no differential effect of young or old serum was observed on the proliferation marker Ki67 at the group level (Fig. 5A). Apoptotic cell death, as measured by cleaved caspase 3 (CC3), increased 2-fold in HPCs cultured with old serum (Fig. 5B-C, *P* = 0.0264). Notably, a subset of serum samples within the old cohort induced marked increases in CC3 expression to 30-40% of total number of cells - values far greater than the mean level of 5.78% cell death induced by young serum. In line with this, the old serum samples that induced the highest levels of cell death also had the lowest cell counts. There was also no differential expression in the levels of neuroblasts as measured by DCX (Fig. 4D) or immature neurons as measured by Map2 (Fig. 5E).

Investigating within the old cohort only, linear and nonlinear regression analyses revealed no relationship between the age of serum donor (69 to 94 yrs) and corresponding effect on apoptotic cell death (Supplementary Fig. 3C) or indeed for any of the other cellular markers measured (Supplementary Fig. 3A-E). Interestingly however, results from the old cohort were more heterogeneous. We show higher variance in the old cohort both for CC3 results (Fig. 5B F test: p<0.0001, F=9.628, DFn=34, DFd=27) and for DCX results (Fig. 5D, F test: p=0.0003, F=4,190, DFn=34, DFd=27), suggesting that variation increases with age possibly owing to a lifetime of varying environmental factors.

### Epidemiological factors and cognition in the older adult cohort contribute to heterogeneity in serum-induced cellular readouts

The older adult cohort in the present study was free from marked clinical cognitive decline and neuropsychiatric conditions as determined by clinical assessment, but many of these persons harboured varied clinical histories including rheumatic, metabolic, cardiovascular and inflammatory conditions (full list and scores in Supplementary Table 1). We next explored whether epidemiological factors contributed to the observed cellular variation following culture with old serum.

The following covariates for the old cohort: age, education (years), gender, score on the Global Deterioration Scale [51], intake of anti-hypertensive medication, pre-existence of inflammatory disease (e.g. arthritis), intake of statins, alongside corresponding cellular readouts (Ki67, CC3, DCX, Map2) following culture with serum, was analysed using multiple regression tools.

**Figure 6.**
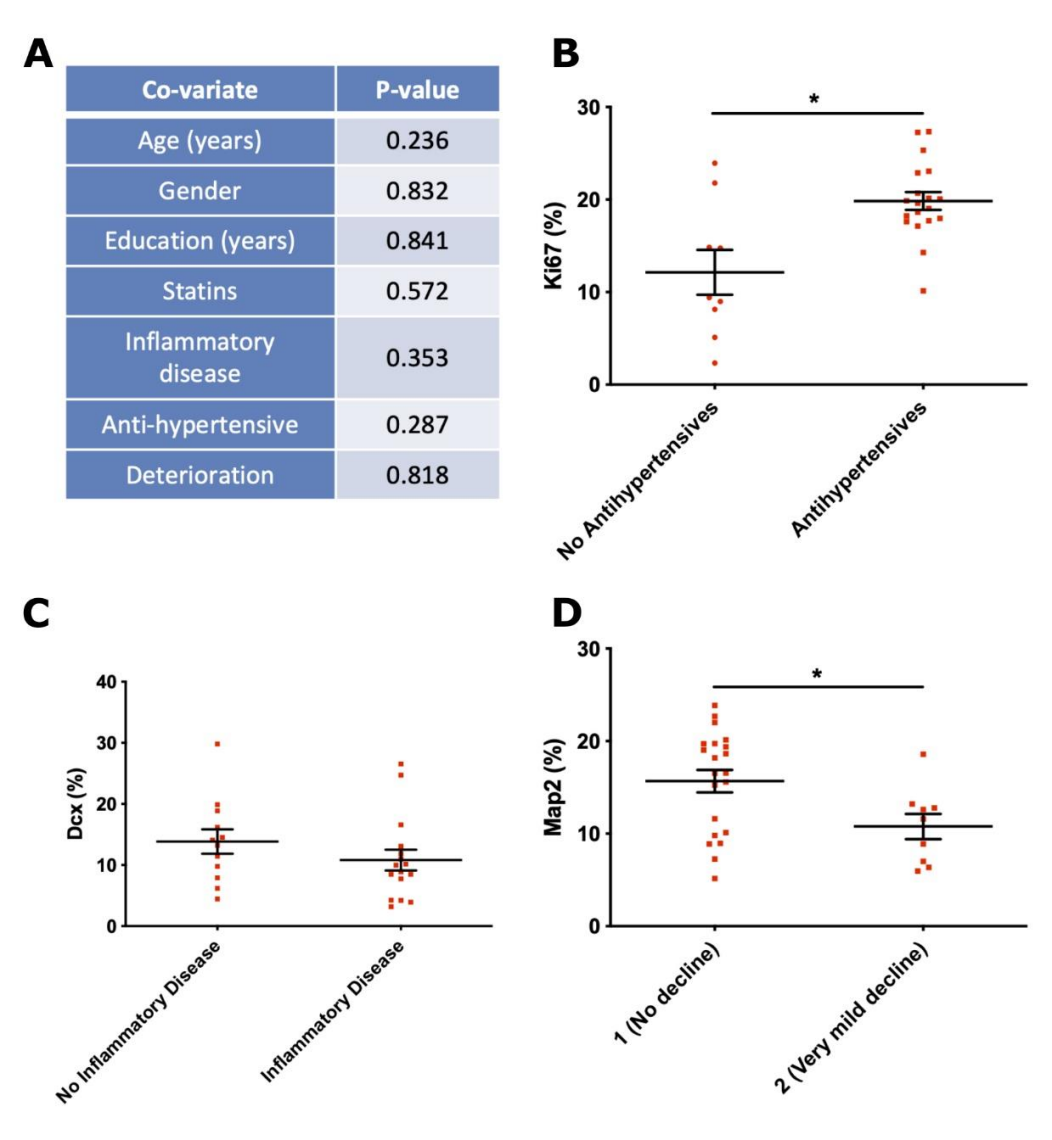
Influence of old cohort covariates on cellular markers. (A) Results from multiple regression analysis evaluating the effects of epidemiological co-variates on the corresponding serum-induced levels of CC3, (B) Ki67 levels compared in context of ongoing antihypertensive medication, (C) DCX levels divided by those with pre-existing or ongoing inflammatory disease, (D) Map2 levels divided by those old subjects showing increased very mild cognitive decline on the global deterioration scale [51]. (B-D) presented as % of total number of cells. n= 3 technical replicates per sample. Unpaired student and Mann-Whitney two-tailed t-tests used as appropriate, *P < 0.05. Error bars = SEM.

Both backward step elimination and combining all covariates into a General Linear Model (GLM) revealed no significant effect of any of these factors in relation to the levels of CC3 (Fig. 6A). For the proliferative marker Ki67, the backward step elimination process revealed that removing the covariate for antihypertensive medication resulted in a less informative model, indicating that the variance in the levels of Ki67 is influenced by whether an old subject is taking antihypertensive medication or not.

This finding was corroborated by plotting this covariate as a single variable against the levels of Ki67, revealing that serum derived from old subjects currently taking antihypertensive medication was linked to a significant increase in the levels of Ki67 (Fig. 6B, P < 0.05). Across the 19 old subjects taking antihypertensive medication, a total of 17 different drugs were being taken (Supplementary Table 2).

For the neuroblast marker Dcx, the backward step elimination process indicated that gender, years of education and the presence of a pre-existing inflammatory disease explained proportions of the variance. Incorporating these 3 covariates into a refined GLM indicated that only inflammatory disease revealed a significant effect on DCX levels (P < 0.05) but this was not corroborated by an analysis of deviance of the refined model, indicating that a large proportion of the variance in DCX levels is still not explained by inflammatory disease (Fig. 6C).

Interestingly, with respect to Map2-positive neurons, the backward step elimination process indicated that both age and deterioration explained proportions of the variance in expression levels. Running Map2 levels as a function of these two co-variates in a refined GLM revealed a significant effect of deterioration (P < 0.05) but not for age (*P* = 0.29). In the GLM, the covariate deterioration divides old subjects based on scores on the Global Deterioration Scale, where a score of 1 indicates no cognitive decline or problems with daily living, whereas a score of two indicates very mild decline such as forgetting the names and locations of objects [51]. In line with the GLM, old individuals with no cognitive decline on the Global Deterioration Scale have significantly higher levels of Map2, when compared to older individuals with very mild cognitive decline (Fig. 6D, P < 0.05).

Together, these findings indicate that epidemiological factors in the old cohort contribute to the variation in serum-induced cellular readouts reported in this study.

**Figure 7.**
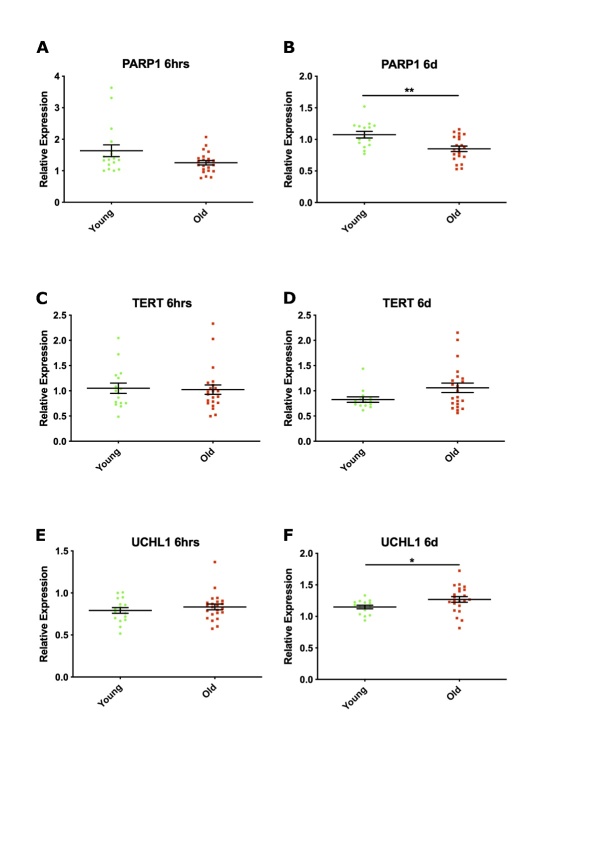
Expression of genes involved in maintaining genomic integrity and proteostasis following culture with young or old serum at 6 hours and 6 days differentiation. Relative expression of (A, B) Poly ADP-ribose polymerase 1 (*PARP1*), (C, D) Telomerase reverse transcriptase (*TERT)* and (E, F) Ubiquitin carboxyl-terminal esterase L1 (UCHL1), normalised to one young subject (21 years), corresponding to 1 on the y axis. Each green circle (young serum, n = 17, mean age of 25.6 years) or red square (old serum, n = 23, mean age of 78 years) represents n =2 technical replicates following analysis of qPCR data, after 6 hrs (hours) or 6 days (d) differentiation of hippocampal progenitors in presence of human serum. Unpaired two-tailed student and Mann-Whitney t-tests as appropriate, ** P < 0.01 error bars = SEM.

### Evaluating molecular hallmarks of ageing in human hippocampal progenitor cells cultured with young and old human serum

A single gene candidate approach was next used to analyse ageing molecular hallmarks (full list in Supplementary Table 3) at the gene expression, protein and post-translational modification level in HPCs treated with young (n=17, mean age of 25.6 years) or old (n=23, mean age of 78 years) human serum (Fig. 2B). These samples represent a subset of the cohort used in the above cellular experiments, which excluded young and old serum linked to divergent effects on apoptosis (Fig. 5B-C) - these samples were used in the whole transcriptome analysis reported in the next set of experiments. Owing to the large number of targets assayed and concerns about multiple testing, the contribution of epidemiological factors in the old cohort to levels of expression of age-linked molecular markers was not considered.

There was no differential change in expression of candidate genes involved with mitochondrial function (NAMPT, DIABLO, DNML1, SOD2, POLG) at 6 hours (Supplementary Fig. 4A-E) or 6 days (Supplementary Fig. 5F-J) of differentiation in the presence of young and old serum. HPCs were also incubated with MitoTracker to label active mitochondria after 7 days of differentiation with human serum. This experiment revealed no differential effect of young or old serum on the levels of active mitochondria (Supplementary Fig. 6A-B).

There was no indication of double-stranded breaks in DNA following culture with young or old serum, as revealed by phosphorylation of histone H2A variant (H2A.X) (Supplementary Fig. 6C-D) or expression of X-ray repair complementing defective repair in Chinese hamster Cells 2 (XRCC2) (Supplementary Fig. 6E-F). Expression of Poly ADP-ribose polymerase 1 (PARP1), indicating increased repair of single stranded DNA breaks, was increased by 40% following 6 hours of culture with young serum (Fig. 7A, *P* = 0.0976), reaching significance at 6 days (Fig. 7B, *P* = 0.0021) when compared to old serum. Prolonged culture with old serum showed a trend of increased expression of telomerase reverse transcriptase (*TERT*) (Fig. 7C-D, *P* = 0.1581) and of the neuron-specific enzyme ubiquitin carboxyl-terminal esterase L1 (*UCHL1*) (Fig. 7E-F, *P* = 0.0485).

There was no differential expression of markers of nutrient sensing pathways (*SIRT1, pSIRT1, ATF4, ATG5, S6, pS6, AMPK, AKT* - (data not shown), cellular senescence (*CDKN2A, CDKN1A, CDKN2B, CDKN2B, TP53* (data not shown), altered intracellular communication (*TNFR1, FADD* - (data not shown)) or NF-κB activation (data not shown) following culture with young and old serum at 6-hour and 6-day time points during the differentiation phase of the assay. Most of the ageing molecular hallmarks evaluated in these experiments were not altered in HPCs exposed to young or old serum. A subset of molecules, however, related to DNA damage (PARP1) and proteostasis (UCHL1) showed significantly altered expression following prolonged culture of HPCs with young or old serum.

### Transcriptome analysis of hippocampal progenitors exposed to young and old serum that induce divergent cell phenotypes

Next, an endophenotype approach was taken and serum samples from subjects from both the young and old cohorts were selected based on the contrasting effects of their serum on apoptotic cell death of HPCs. Differential expression was then assesed via microarray anlysis. The top 20 known genes linked to these probes are listed in Table 1.

**Table 1:** Top 20 genes differentially expressed in response to young and old serum.

Gene	Name	*P* value
*ISCA1*	Iron-sulfur cluster assembly	0.000065
*ANXA2*	Annexin A2	0.00014
*ATP6AP1*	ATPase, H+ transporting, lysosomal accessory protein 1	0.0004
*ARPP19*	cAMP-regulated phosphoprotein 19	0.0004
*PFTK1*	Serine/threonine-protein kinase PFTAIRE-1	0.00049881
*TUBB*	Tubulin, beta	0.0006
*ASAP2*	ArfGAP with SH3 domain, ankyrin repeat and PH domain 2	0.001035752
*PPAP2A*	Phosphatidic acid phosphatase type 2A	0.00107
*ABTB1*	Ankyrin repeat and BTB (POZ) domain containing 1	0.001171827
*TMEM149*	Transmembrane protein 149	0.001187346
*ZC4H2*	Zinc finger, C4H2 domain containing	0.001198645
*C3ORF72*	Chromosome 3 open reading frame 72	0.001283206
*ZNF264*	Zinc finger protein 264	0.0013
*IFIT3*	Interferon-induced protein with tetratricopeptide repeats 3	0.0013
*ZMYM2*	Zinc finger, MYM-type 2	0.0013
*ZDHHC6*	Zinc finger, DHHC-type containing 6	0.001367863
*ENDOG*	Endonuclease G	0.0017
*OTUD4*	OTU domain containing 4	0.001696685
*PRDX3*	Peroxiredoxin 3	0.001814213
*TRIM45*	TRIM45 tripartite motif-containing 45	0.001920272

141 probes reached a significance threshold *P* value of < 0.01 (uncorrected for multiple comparisons) for differential expression at one or more of the 5 time-points (1, 6, 24, 72 and 144 hours) following exposure to young or old serum.

**Figure 8.**
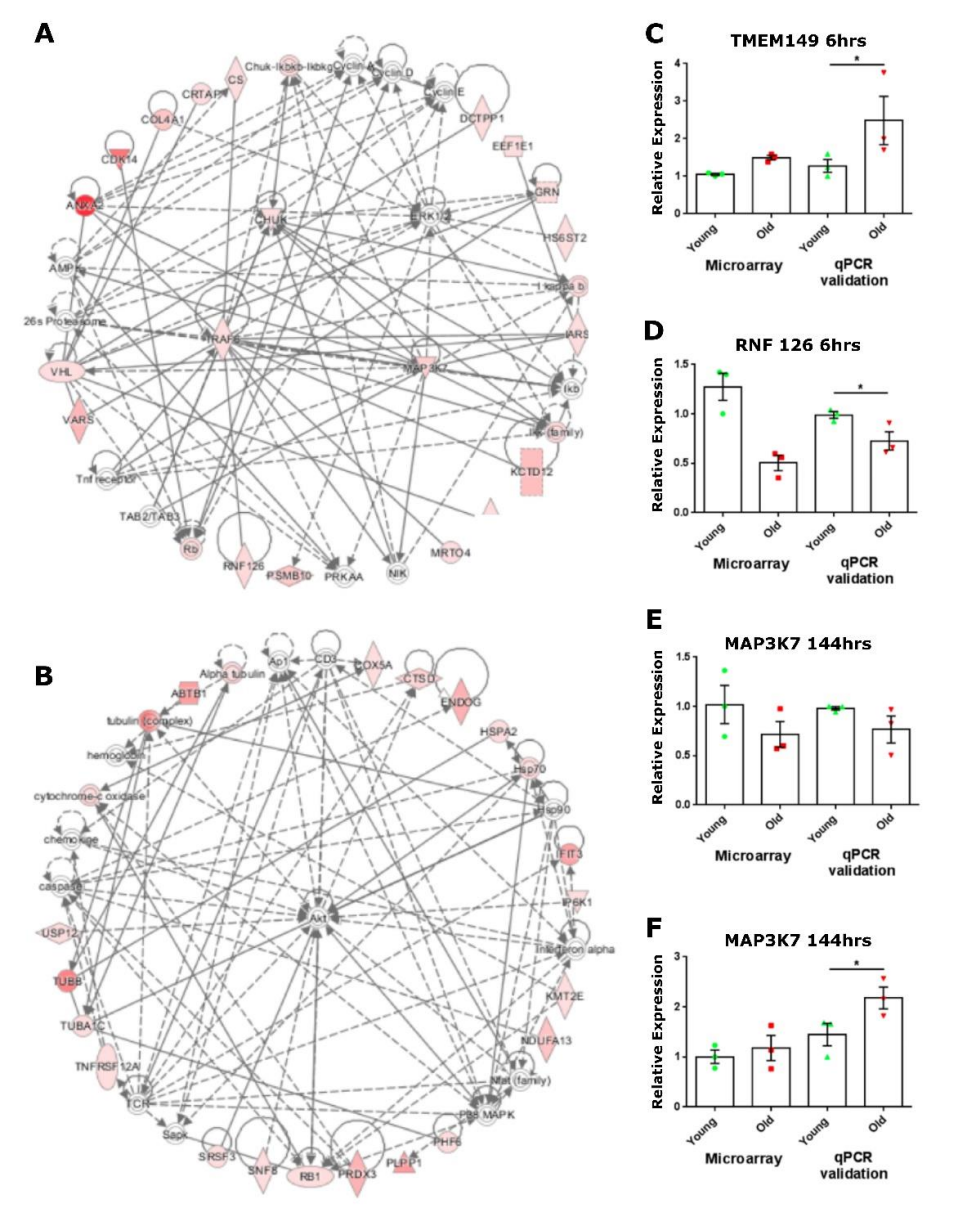
Validation of differentially expressed genes in response to serum from older adults. (A, B) Networks formed by differentially expressed genes in response to young and old serum. 111 differentially expressed genes during differentiation of hippocampal progenitors in the presence of young or old serum were analysed using Ingenuity Pathway Analysis (IPA) to assess network relationships between candidate genes, A) top network associated functions included “System Development and Function” and “Cell to Cell Signalling”, B) second highest network associated functions included “Cellular Development”, “Cellular Growth and Proliferation” and “Cancer”. (A, B) significance of genes reflected on continuous colour scale from red (most significant) to light pink (least significant). Inferred molecular interactions identified by IPA are shown in grey. (C-F) Validation by qPCR of differentially expressed microarray candidate genes in response to young or old serum. Relative expression of microarray candidates C) transmembrane 149 (*TMEM149*) D) ring finger protein 126 (RNF126) E) mitogen-activated protein kinase 7 (*MAP3K7*) F) endonuclease G (*ENDOG*) normalised to one young-serum induced readout, corresponding to 1 on the y axis for both the microarray and qPCR validation. Each green circle (young, n = 3) or red square (old, n = 3) represents expression values during differentiation of hippocampal progenitors in the presence of human serum at the stated time point. Microarray statistics not included. Unpaired student one-tailed t-test conducted on qPCR data, * P < 0.05, error bars = SEM.

Network analysis of this gene list revealed a top scoring network with associated functions linked to “System Development and Function” and “Cell-to-Cell Signalling” (*P* < 0.05). The central node of this network was TNF receptor-associated 6 (TRAF6) (Fig. 8A). The serine/threonine kinase ERK1/2 was implicated in the top scoring network, as several candidates interacted with this kinase (Fig. 8A). The second highest scoring network centred on “Cellular Development”, “Cellular Growth and Proliferation” and “Cancer”. Differentially expressed genes in this network show multiple relationships with the central node AKT (Fig. 8B).

Given that the candidate probes generated by the young *versus* old analysis did not survive correction for multiple comparisons, a common outcome in microarray analysis owing to the stringency of methods used, validation of these candidates by alternative experimental methods was required to rule out false positives [52]. Candidates were selected for validation by qPCR based on the strength of their *P* value, implication by pathway analysis and novelty, in having no previously demonstrated link to neural stem cell function, ageing or the heterochronic parabiosis literature.

In line with the findings from the microarray, validation by qPCR revealed a 2-fold increase in expression of transmembrane protein 149 (*TMEM149*), also known as IGF-like family receptor 1 (IGFLR1), in HPCs after 6 hours of differentiation in the presence of old serum, when compared to cells treated with young serum (Fig. 8A, *P* = 0.05). Ring finger protein 126 (*RNF126*) is an E3 ubiquitin-protein ligase involved in preparing substrates for the ubiquitin-proteasome system. The array revealed that at the 6-hour differentiation time point, young serum increased HPCs expression of RNF126 by 75% (Supplementary Table 4, *P* = 0.0042) and this was corroborated by qPCR validation (Fig. 8B, *P* = 0.0042). The array revealed that *MAP3K7* expression was increased by 30% in HPCs treated with young serum at 144 hours into the differentiation phase of the assay (Supplementary Table 4, *P* = 0.0032) and both the direction and magnitude of this expression was observed during validation (Fig. 8C, *P* = 0.0975). The array also revealed that expression of endonuclease (ENDOG), an enzyme required for mitochondrial replication and caspase-independent apoptosis, was increased by old serum at the 144-hour time point (Table 1, *P* = 0.0012), and this result was validated by qPCR where old serum increased ENDOG expression by 80% when compared to young serum (Fig. 8D, *P* = 0.039).

The expression profiles of the validated candidate genes *TMEM149, RNF126, MAP3K7* and *ENDOG* were next measured in HPCs exposed to young and old serum samples not linked to divergent apoptotic responses. At 6 hrs differentiation, the expression of *TMEM149* or *RNF126* was similar in HPCs exposed to young or old serum (Supplementary Fig. 6E-F) in contrast to the differential effect elicited by the serum samples used in the microarray experiments (Fig. 8A-B). Similarly, both the levels of *MAP3K7* and *ENDOG* at the 6-day time point were unchanged by young or old serum, whereas the samples used in the array significantly altered expression of these genes (Fig. 8C-D).

Annexin 2 (ANXA2), cytochrome c oxidase subunit Va (COX5A), citrate synthase (CS) and conserved helix-loop-helix ubiquitous kinase (CHUK) were candidate genes revealed by the microarray (Table 1 and Fig. 2A). Their differential expression in HPCs following culture with young or old serum was not validated by qPCR (Supplementary Fig. 6A-D), identifying these candidates as false positives. Together, technical and biological validation of the microarray study by qPCR has affirmed the differential expression of several candidate genes in differentiating HPCs in the presence of serum.

## DISCUSSION

Using a novel *in vitro* assay, we evaluated the impact of the ageing human milieu on human HPCs. We show that *in vitro* assay results are associated to *in vivo* phenotypes and demonstrate that the ageing human systemic milieu has a detrimental effect on the cellular viability of human HPCs. In addition, our results show that the negative effects of the ageing human milieu, are characterised by marked variation that is partly attributable to the divergence of epidemiological factors among older subjects.

Using the MATO cohort, we first gained insight into the translatability of this novel *in vitro* model. Our results showing the predictive effects of age on hippocampal volumes are in line with several studies reporting an age-related reduction in hippocampal subfield volumes [53-55]. This reduction is often attributed to neuronal and dendritic loss but the exact mechanisms behind this phenomenon remain unclear [56]. Others have highlighted contradictory results when investigating age-related changes in hippocampal subfield volumes, with some reporting no evidence of association and others reporting associations limited to specific subfields [56-59]. Until recently, inadequacies in imaging resolution and available techniques have prevented accurate segmentation of the subfields and have contributed to discrepancies between studies [60]. Results in the present study were obtained using the latest FreeSurfer algorithm which ensures accurate segmentation and allows for the novel separation of the CA3, CA4 and DG subfields providing stronger support for the existing evidence of an age-related decline in hippocampal volume [60, 61].

Importantly, we also show associations between two key markers of *in vitro* stem cell health (neuroblast number and DNA damage) and hippocampal volumes following the serum assay. Though these associations did not survive correction for multiple testing, and they require confirmation by further research and larger samples, they support a biologically relevant role for the systemic milieu and a function as a likely reflection of brain health.

The associations between neuroblast number and hippocampal volumes are of particular interest given recent results by Powell and colleagues. Using the same HPCs line used here, they showed that differentiation causes the upregulation of a gene set that was significantly enriched for genes predictive of hippocampal volume [42]. These results suggest similar mechanisms are involved in both the regulation of the differentiation process and in the determination of hippocampal volumes. This is in line with our data showing a positive association between neuroblast number and hippocampal volumes. It is also interesting to note that an association with larger subfield volumes is supportive of the possible effect of neurogenesis on brain volume [56]. Further studies assessing the maturation and integration potential of the neuroblasts and resulting neurons, however, would be needed to support this claim.

Despite these promising results, experiments with serum from MATO cohort participants showed no association between cellular marker and donor age. This was surprising given the rodent literature suggesting an ageing systemic environment causes alterations in hippocampal neural stem cells [3, 8, 9]. In this cohort, participant age ranged from 52 to 89, suggesting either that the effects of an ageing systemic environment plateaus off past middle-age or that wider age differences are necessary to detect these effects. Given the large interindividual variability typically seen in human cohorts, we explored whether repeating these experiments using a cohort with distinct young and old groups would show associations between donor age and cellular readouts. Indeed, using the young and old cohort, we demonstrate that serum derived from old individuals induced a 2-fold increase in apoptotic cell death of HPCs when compared to the effects of young serum. This work recapitulates aspects of the negative impacts of the ageing milieu on neural stem cell viability in rodents as revealed by heterochronic parabiosis experiments[8, 9].

In the present study, however, there was no differential effect of young vs. old serum on markers of HPCs proliferation or differentiation contrasting the results obtained in the parabiosis literature [3, 5, 9]. Possible reasons for this discrepancy include differences in experimental design and increased variability in human cohorts. For example, parabiosis experiments typically last for 4-5 weeks reflecting long-term exposure to the young and old milieu. In addition, intraventricular administration of old rodent plasma occurred 4 times over a 10-day period [9]. These experimental designs involve extended (parabiosis) or repeated (intraventricular administration) periods of exposure to the ageing milieu and thus differ from the shorter length and type of exposure employed in the present study. As such, it is possible that prolonged and repeated exposure is required for the negative effect of the ageing milieu to manifest in altered expression of HPCs markers of proliferation and differentiation. Indeed, our findings show that some molecular hallmarks of ageing (PARP1, TERT and UCHL1) are increased following prolonged culture with old serum but many markers remain unchanged, suggesting longer exposures are required to induce an ageing phenotype.

Increased genetic and environmental variability in humans, when compared to the effects of a group of inbred mice - derived from the same genetic background and reared in homogenous environmental conditions - are crucial considerations when comparing the present findings to previous work using rodent systems. In the present study, several markers were characterised by marked variability following exposure to old human serum, suggesting that environmental exposures influence the impact of the human milieu on stem cell fate commitment. Indeed, our results show that use of antihypertensive medication is linked to increased proliferation and very mild cognitive deterioration associated with reduced Map2 levels. Taken together, this indicates that the evaluation of the ageing human systemic environment upon human stem cell function must incorporate environmental, demographic and clinical covariates into multi-regression analyses.

The differences between the present findings and previous parabiosis studies may also be attributable to the different ages employed and the species differences in the temporal dynamics of neurogenesis. The young human cohort ranged from age 21 to 38 years, and the old cohort comprised of 68- to 94-year-olds, whereas Villeda and colleagues used groups of mice aged 2-4 and 18-22 months for young and old groups, respectively. Therefore, no serum samples in our study correspond to the youngest mice used in the parabiosis experiments. Further to this, the rate of decline in human hippocampal neurogenesis appears less pronounced than that observed in mice, revealing a species difference [62]. Perhaps in humans, the largest changes occur in childhood and adolescence, with only a gentle decrease thereafter. In human mid to late life therefore, there is both a shallow slope of decline and increasing variability through environmental exposures. As rodents do not have a prolonged post-reproductive life it is difficult to compare the human middle to late life period with respective rodent data.

This suggests that a larger cohort with more detailed information on environmental exposures and younger participants may be required to fully evaluate differences in the impact of the ageing milieu on HPC function. Indeed, it has been shown that umbilical cord plasma from humans is particularly effective in revitalising the hippocampus and improving cognitive function in aged mice [63].

Though expression of many of the molecular hallmarks of ageing was unchanged following exposure to young and old human serum, a subset of hallmarks, related to DNA damage (PARP1) and proteostasis (UCHL1) were altered by prolonged culture of HPCs with old serum (Table 2). Importantly, our study also highlights novel candidate genes (*RNF126* and *ENDOG*) and shows expression alterations that are largely in line with existing literature as seen in Table 2. Furthermore, we highlight mitochondrial function as an important mechanism by showing increased levels of the mitochondrial endonuclease (ENDOG) in response to treatment with old serum that was linked to high levels of apoptosis. With age, the efficacy of the mitochondrial respiratory chain diminishes, leading to increased electron leakage and reduced ATP generation [64]. Together these data suggest that old serum induces mitochondrial-linked cellular dysfunction and that cell death arising from this might be prevented by manipulating the levels of ENDOG - possibly by modulating environmental exposures such as diet.

**Table 2:** Table showing key genes identified in this study in relation to aging literature.

Gene	Current Study	Existing Literature
*PARP1*	Decrease in response to old serum (6 days timepoint, qPCR)	PARP1 is overactivated in aging and age-related disease, inhibition of PARP1 was shown to be beneficial in delaying progression [65-67].
*UCHL*	Increase in response to old serum (6 days timepoint, qPCR)	An increase in UCHL was shown in aging mice. In addition, plasma UCHL levels showed a positive correlation with Parkinson’s disease severity in a recent case-control study [68, 69].
*TMEM149*	Increase in response to old serum (6-hour timepoint, identified via microarray, validated by qPCR)	Role of TMEM149 in aging remains largely unexplored. However, a recent longitudinal multiomics profiling study in healthy individuals showed a positive correlation between TMEM149 and age [70].
*RFN126*	Decrease in response to old serum (6-hour timepoint, identified via microarray, validated by qPCR)	RNF126 is required for Bag6-dependent ubiquitination and has been shown to initiate degradation of mislocalised and misfolded proteins. This suggests a decrease in RNF126 may be associted to an accumulation of mislocalised and misfolded proteins [71, 72].
*ENDOG*	Increase in response to old serum (6-day timepoint, identified via microarray, validated by qPCR)	Higher levels of EndoG were found in whole brain lystaes of 34-week-old mice when compared with 5-week-old mice. In addition, nuclear levels of ENGOG were 153% higher in the aged rat plantaris at 24 months, when compared to younger counterparts aged 6 months [73, 74].

The establishment of the *in vitro* serum assay presented here has been used to show that the ageing human systemic milieu has a detrimental effect on the viability of human HPCs. This finding recapitulates aspects of the inhibitory effect of the ageing mouse milieu on neural stem cell function. We also show that an endophenotype approach - based on cellular readouts from our assay of hippocampal neurogenesis - can be used to better evaluate ageing molecular phenotypes following culture with human serum from an ageing and epidemiologically diverse cohort. Importantly, we show that although key *in vitro* readouts following the cellular assay correlate with *in vivo* phenotypes such as hippocampal volumes and cognitive performance, they do not show association to chronological age. This supports the notion that a lifetime of contributing factors causes increased divergence between chronological and (neuro)biological age which leads to greater heterogeneity in older populations. Our findings support the here-described serum assay as a potential biomarker for neuorbiological age, making it a valuble tool for studies investigating ageing and age-related conditions as well as lifestyle factors such as diet and exercise.

Future studies using this model should investigate divergent cellular and molecular responses to serum derived from cohorts defined by epidemiological, environmental and clinical factors, with the goal of using these responses to determine key molecular pathways responsible for ageing’s heterogeneity and to aid discovery of biomarkers of biological age. In particular, future studies aimed at identifying the serum components responsible for these beneficial or detrimental effects could provide unprecedented insight into the mechanisms of ageing.

## Supplementary Materials

The Supplemenantry data can be found online at: www.aginganddisease.org/EN/10.14336/AD.2021.0409.


