# Supplementary material for: Serum from Older Adults Increases Apoptosis and Molecular Aging Markers in Human Hippocampal Progenitor Cells
Source: Aging Dis. 2021 Dec 1;12(8):2151–72. doi: 10.14336/AD.2021.0409 (PMC8612606; doi:10.14336/AD.2021.0409)
Supplement: Supplementary file 1 [file AD-12-8-2151-s.pdf]

SUPPLEMENTARY DATA

Serum from Older Adults Increases Apoptosis and Molecular Ageing Markers in Human Hippocampal Progenitor Cells

Chiara de Lucia[1,#], Tytus Murphy[1,#], Aleksandra Maruszak[1], Paul Wright[1], Timothy R. Powell[2], Naomi Hartopp[1], Simone de Jong[2], Michael J O'Sullivan[1, 3], Gerome Breen[2], Jack Price[1], Simon Lovestone[4], Sandrine Thuret[1*]

© 2021. De Lucia C et al. Published online at http://www.aginganddisease.org/EN/10.14336/AD.2021.0409

SUPPLEMENTARY DATA

Supplementary Figures

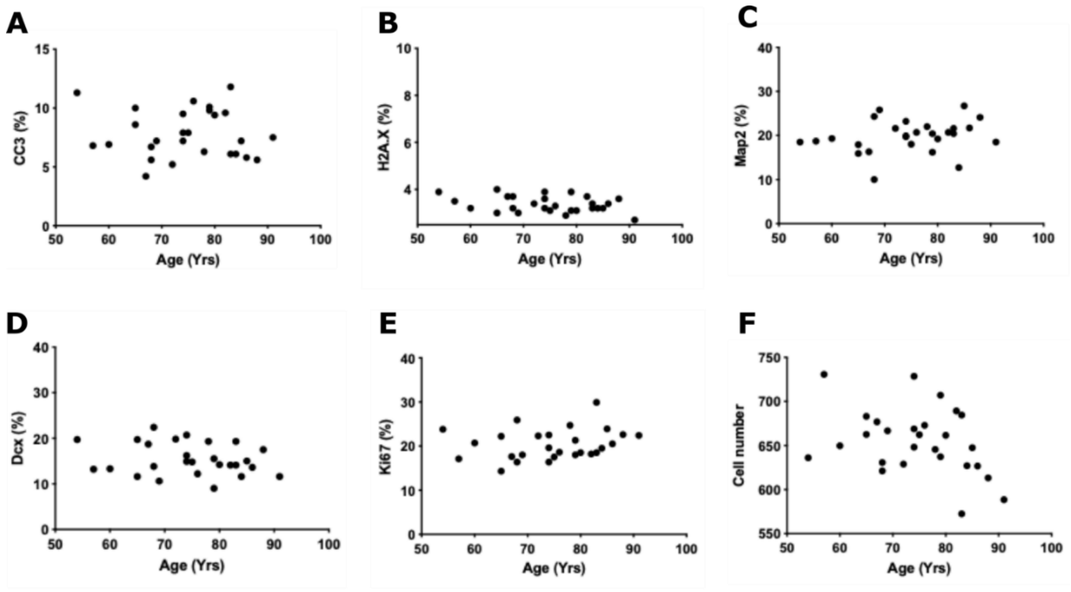

Cellular readouts	r	p value
Age and CC3%	-0.05	0.82
Age and H2AX%	-0.26	0.20
Age and MAP2%	0.31	0.12
Age and DCX%	-0.18	0.36
Age and KI67%	0.28	0.16
Age and Cells per Field	-0.36	0.06

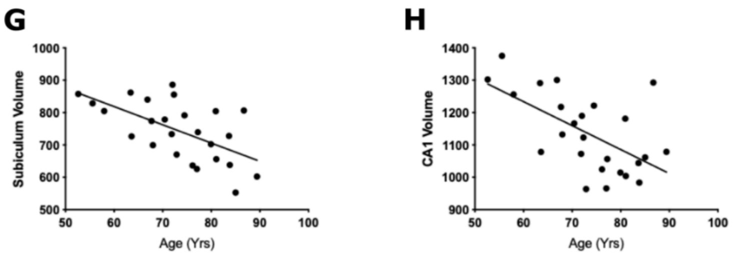

Age and Subfields Volumes	r	r^2	p value
**Age and Subiculum**	**-0.59**	**0.36**	**0.002***
**Age and CA1**	**-0.78**	**0.43**	**<0.0001***

Supplementary Figure 1. (A-F) Effect of MATOC serum donor's age on cellular markers. Scatterplots showing the percentage of positive HPC0A07/03A cells following the differentiation assay, for each cellular marker following serum treatment and the corresponding participant's age. Markers for apoptosis (CC3), DNA damage (H2AX), immature neurons (MAP2), neuroblasts (DCX) and proliferation (Ki67) are reported. Also reported is the number of cells per field as a measure of cell density based on the nuclear marker DAPI. Each dot represents a participant. Table reports correlation results of the effect of serum donor's age on cellular markers. r value and p value of each Spearman's correlation assessing the association between each cellular marker and serum donor's age**. (G-H) Effect of age on hippocampal subfield volume**. Scatterplots showing linear regressions assessing the relationship between participant's age in years and hippocampal subfields **(G)** Subiculum, **(H)** CA1. Liner regression line is shown on each graph. Correlation and linear regression results of the effect of age on hippocampal subfield volumes. Table shows the results of correlations and linear regressions testing the association between age and hippocampal volumes. r value following Spearman's correlation and the r2 and p values following linear regressions are reported. Following Bonferroni correction to account for the 6 multiple comparisons, the significance threshold for this analysis was set to 0.0083. Significant results before correction are in bold. * denotes results surviving multiple testing correction.

© 2021. De Lucia C et al. Published online at http://www.aginganddisease.org/EN/10.14336/AD.2021.0409

SUPPLEMENTARY DATA

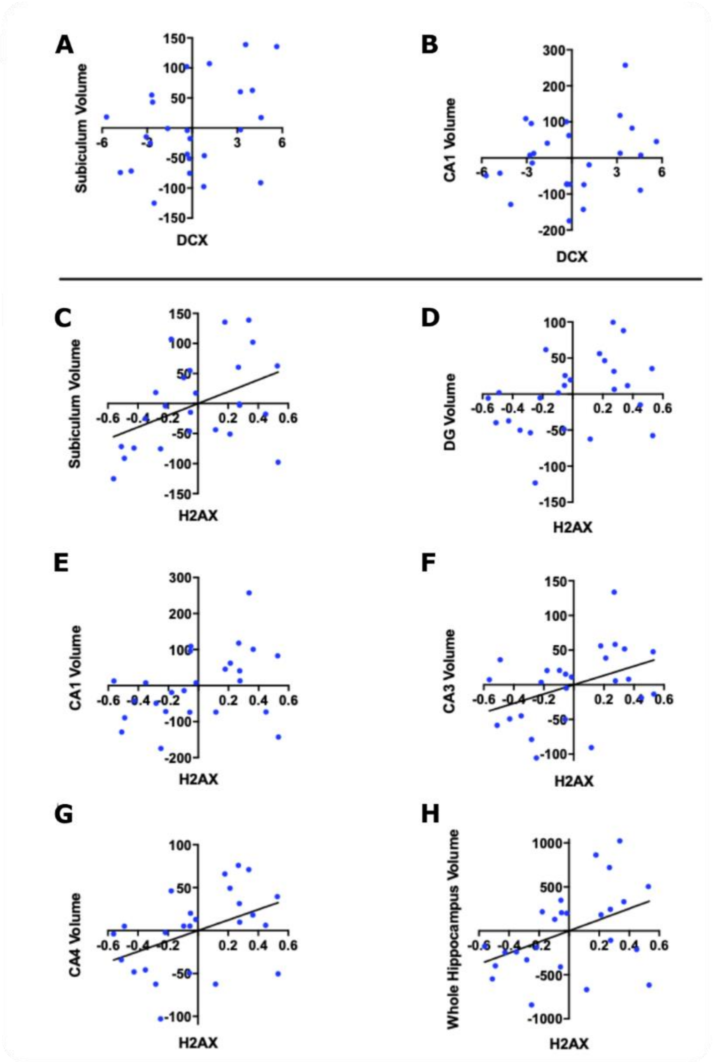

DCX and Subfield Volumes	r	r^2	p value
DCX and Subiculum	0.28	0.14	0.065
DCX and CA1	0.10	0.06	0.258
**H2AX and Subfield Volumes**	**r**	**r^2**	**p value**
**H2AX and Subiculum**	**0.43**	**0.20**	**0.025**
H2AX and Dentate Gyrus (DG)	0.38	0.14	0.061
H2AX and CA1	0.41	0.15	0.053
**H2AX and CA3**	**0.39**	**0.17**	**0.039**
**H2AX and CA4**	**0.49**	**0.19**	**0.029**
**H2AX and Whole Hippocampus**	**0.45**	**0.19**	**0.028**

Supplementary Figure 2. (A-B) Associations of DNA damage marker to hippocampal subfield volumes. 1 Scatterplots showing non-significant associations between neuroblasts (DCX expression) following serum incubation and hippocampal volumes. In each graph, the x-axis shows the age-regressed percentage of HPC0A07/03A cells positive for DCX expression, and the y-axis shows the age-regressed volume of the specific hippocampal subfields **(A)** Subiculum **(B)** CA1. **(C-H) Scatterplots showing the association between DNA damage (H2AX expression) following serum incubation and serum donor's hippocampal volumes.** In each graph, the x-axis shows the age-regressed percentage of HPC0A07/03A cells positive for H2AX expression, and the y-axis shows the age-regressed volume of the specific hippocampal subfield. **(A)** Subiculum, **(B)** Dentate Gyrus (DG), **(C)** CA1, **(D)** CA1, **(E)** CA4, **(F)** Whole hippocampus Linear regression line shown in black, graphs with no line indicate non-significant correlation results. **Table** showing correlation and linear regression results for the association of DCX / H2AX to hippocampal subfield volumes. r value following Spearman's correlation and the r2 and p values following linear regressions are reported. Bonferroni correction was used to account for 36 multiple comparisons (6 subfields and 6 markers), the significance threshold for this analysis was set to 0.0014. Significant results before correction are in bold. No association survived multiple testing correction.

© 2021. De Lucia C et al. Published online at http://www.aginganddisease.org/EN/10.14336/AD.2021.0409

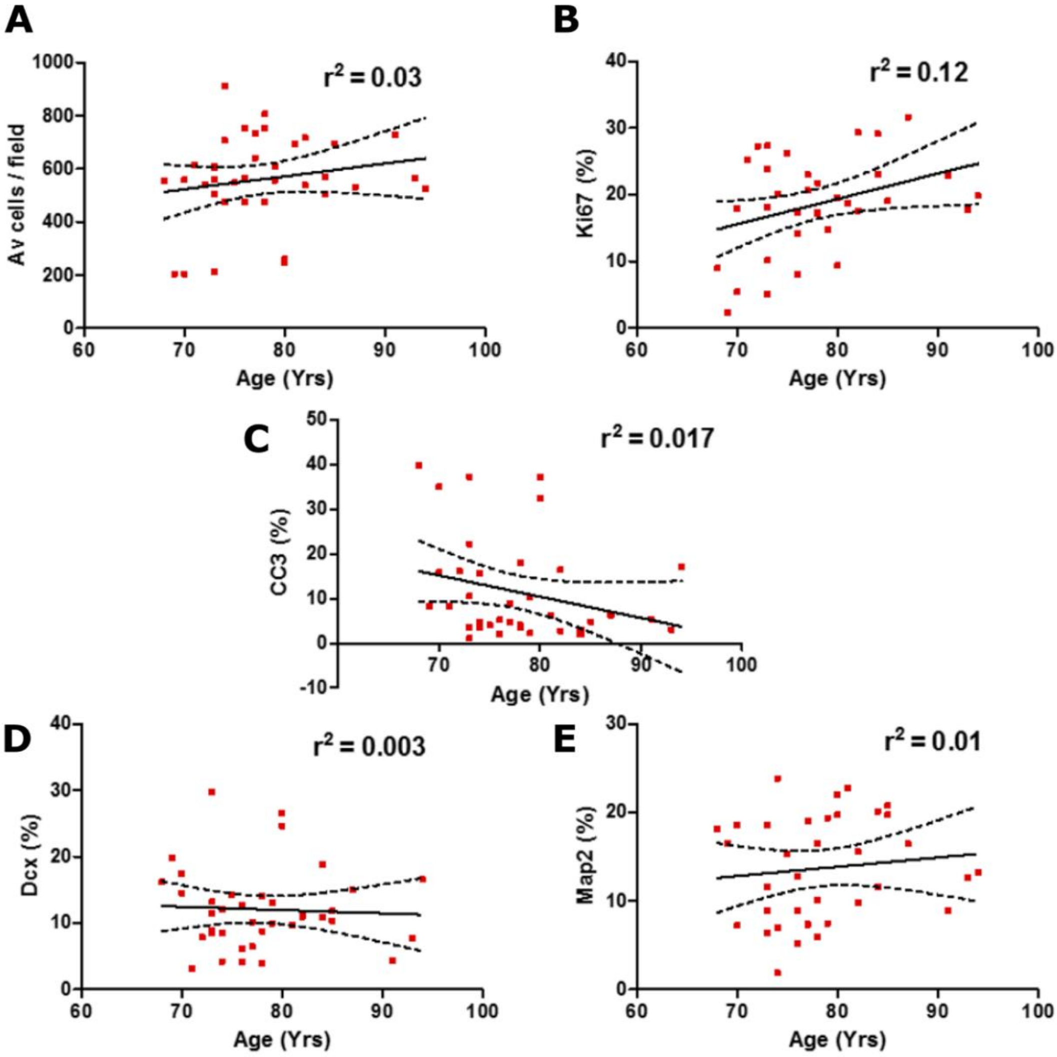

Supplementary Figure 3. Relationship between age of serum donor and cellular read-outs in the old cohort: (A) Total number of hippocampal progenitor cells divided by fields counted **(B)** proliferation, Ki67 (%), **(C)** apoptotic cell death, CC3 **(D)** neuroblasts, DCX (%) and **(E)** immature neurons, Map2 (%) following 9 days of progenitor cell culture with old (red squares) human serum plotted against age of subject. **B**, **C**, **D** and **E** presented as % of total number of hippocampal progenitor cells, n= 3 technical replicates per sample. Linear and non-linear regression analysis undertaken and values of r^2 presented, dashed lines reflect 95% confidence intervals.

© 2021. De Lucia C et al. Published online at http://www.aginganddisease.org/EN/10.14336/AD.2021.0409

SUPPLEMENTARY DATA

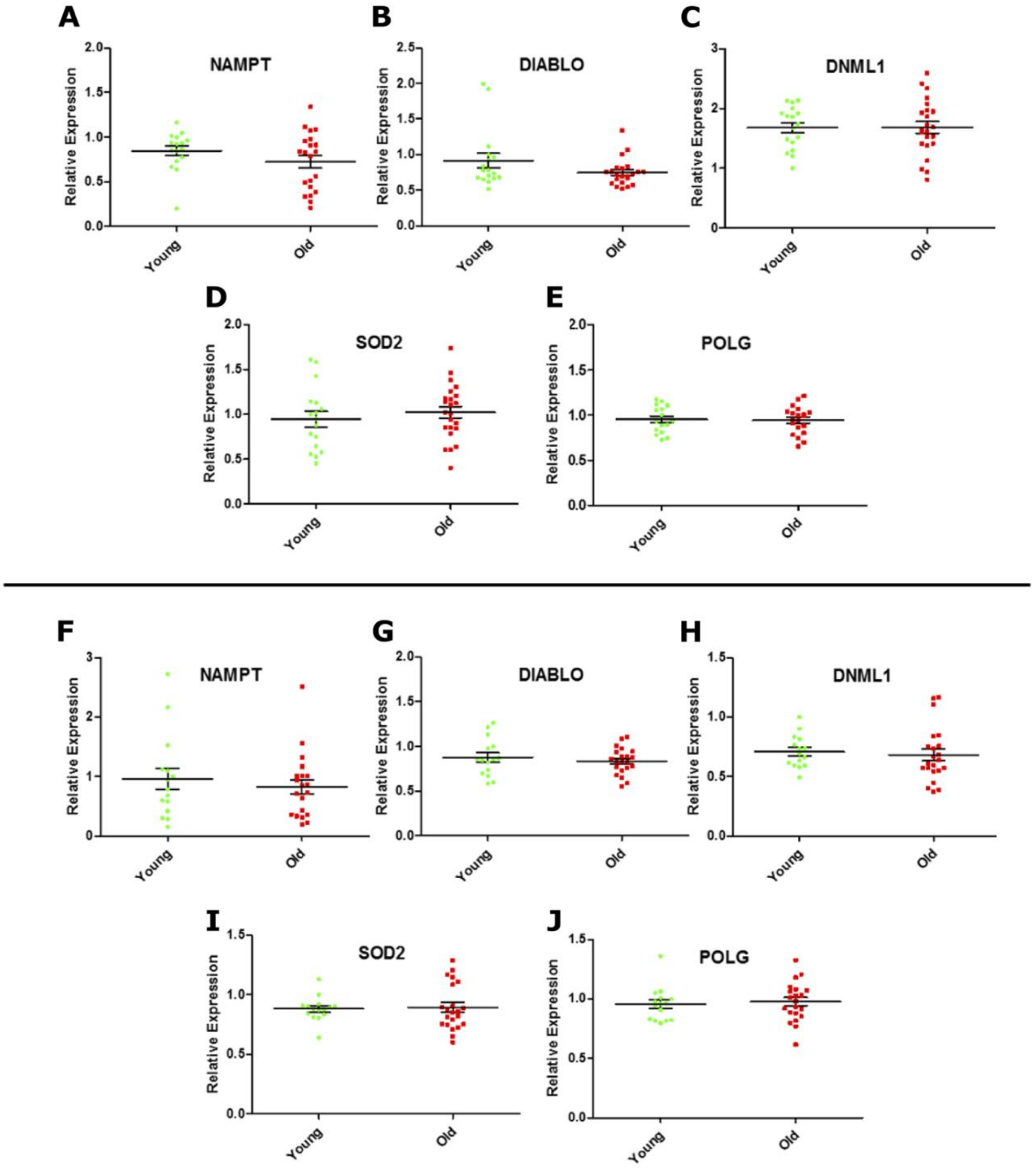

Supplementary Figure 4. (A-E) Expression of genes involved in mitochondrial function in response to young or old serum at 6 hours differentiation: relative expression of **(A)** Nicotinamide phosphoribosyltransferase (NAMPT) **(B)** DIABLO **(C)** Dynamin 1-like (DNML1) **(D)** Superoxide dismutase 2 (SOD2) and **(E)** DNA polymerase subunit gamma (POLG) normalised to one young subject (21 years), corresponding to 1 on the y axis. Each green circle (young serum, n = 17, mean age of 25.6 years) or red square (old serum, n = 23, mean age of 78 years) represents n = 2 technical replicates following analysis of qPCR data, after 6 hours differentiation of hippocampal progenitors in presence of human serum. Unpaired student and Mann Whitney t-tests as appropriate, error bars = SEM. **(F-J) Expression of genes involved in mitochondrial function in response to young or old serum at 6 days differentiation:** relative expression of **(F)** Nicotinamide phosphoribosyltransferase (NAMPT) **(G)** DIABLO **(H)** Dynamin 1-like (DNML1) **(I)** Superoxide dismutase 2 (SOD2) and **(J)** DNA polymerase subunit gamma (POLG) normalised to one young subject (21 years), corresponding to 1 on the y axis. Each green circle (young serum, n = 17, mean age of 25.6 years) or red square (old serum, n = 23, mean age of 78 years) represents n = 2 technical replicates following analysis of qPCR data, after 6 days differentiation of hippocampal progenitors in presence of human serum. Unpaired student and Mann Whitney two-tailed t-tests as appropriate, error bars = SEM.

© 2021. De Lucia C et al. Published online at http://www.aginganddisease.org/EN/10.14336/AD.2021.0409

SUPPLEMENTARY DATA

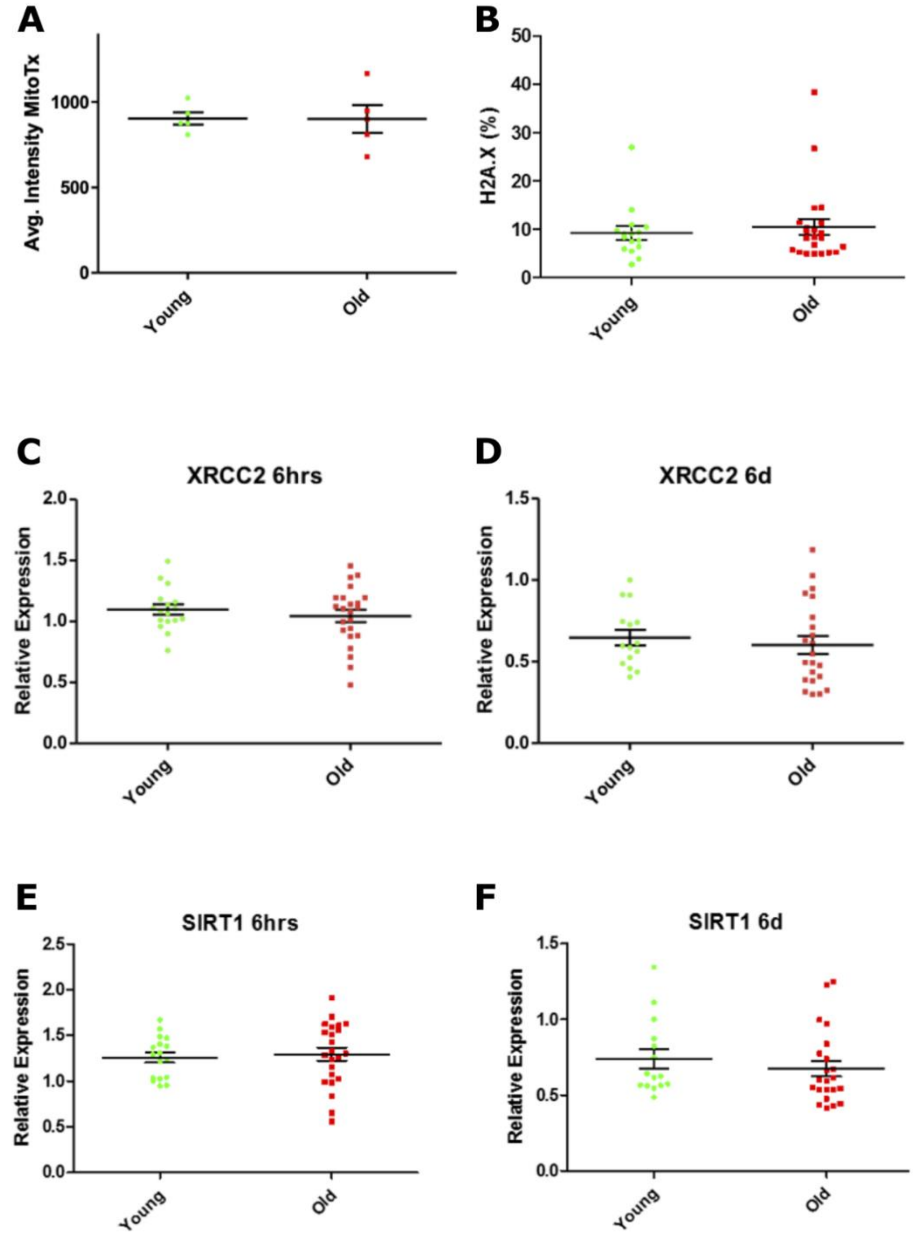

Supplementary Figure 5. (A) There is no change in the levels of active mitochondria in response to young or old serum: the levels of active mitochondria in hippocampal progenitor cells were assessed by quantifying the average intensity of MitoTracker (MitoTx) labelling following 7 days of differentiation in the presence of young (n = 5, green circle) or old (n = 5, red square) serum. Increased intensity reflects increased numbers of active mitochondria. n = 3, unpaired student and Mann Whitney two-tailed t-tests as appropriate, error bars = SEM. **(B) The DNA damage marker H2A.X is not altered in response to young or old serum:** H2A.X presented as a % of total number of cells in hippocampal progenitor cells following 7 days of the differentiation in the presence of young (green dot, n = 17, mean age of 25.6 years) or old (red square, n = 23, mean age of 78 years) serum. n = 3 technical replicates per samples, unpaired student and Mann Whitney t-tests as appropriate, error bars = SEM. **(C-D) Expression of genes involved in maintaining genomic integrity following culture with young or old serum at 6 hours and 6 days differentiation:** relative expression of X-ray repair complementing defective repair in Chinese hamster Cells 2 (XRCC2) normalised to one young subject (21 years), corresponding to 1 on the y axis. Each green circle (young serum, n = 17, mean age of 25.6 years) or red square (old serum, n = 23, mean age of 78 years) represents n =2 technical replicates following analysis of qPCR data, after 6 hrs (hours) **(C)** or 6 days **(D)** differentiation of hippocampal progenitors in presence of human serum. Unpaired two-tailed student and Mann-Whitney t-tests as appropriate, ** $P < 0.01$ error bars = SEM. X. **(E-F) Expression of SIRT1 following culture with young or old serum at 6 hours and 6 days differentiation:** relative expression of SIRT1 at **(E)** 6 hours (hrs) and **(F)** 6 days (d) normalised to one young subject (21 years), corresponding to 1 on the y axis. Each green circle (young serum, n = 17, mean age of 25.6 years) or red square (old serum, n = 23, mean age of 78 years) represents n = 2 technical replicates following analysis of qPCR data, after 6 hrs (hours) or 6 days (d) differentiation of hippocampal progenitors in the presence of human serum. SIRT1 = Silent mating type information regulation 2 homolog 1. Unpaired student and Mann-Whitney two tailed t-tests as appropriate, error bars = SEM.

© 2021. De Lucia C et al. Published online at http://www.aginganddisease.org/EN/10.14336/AD.2021.0409

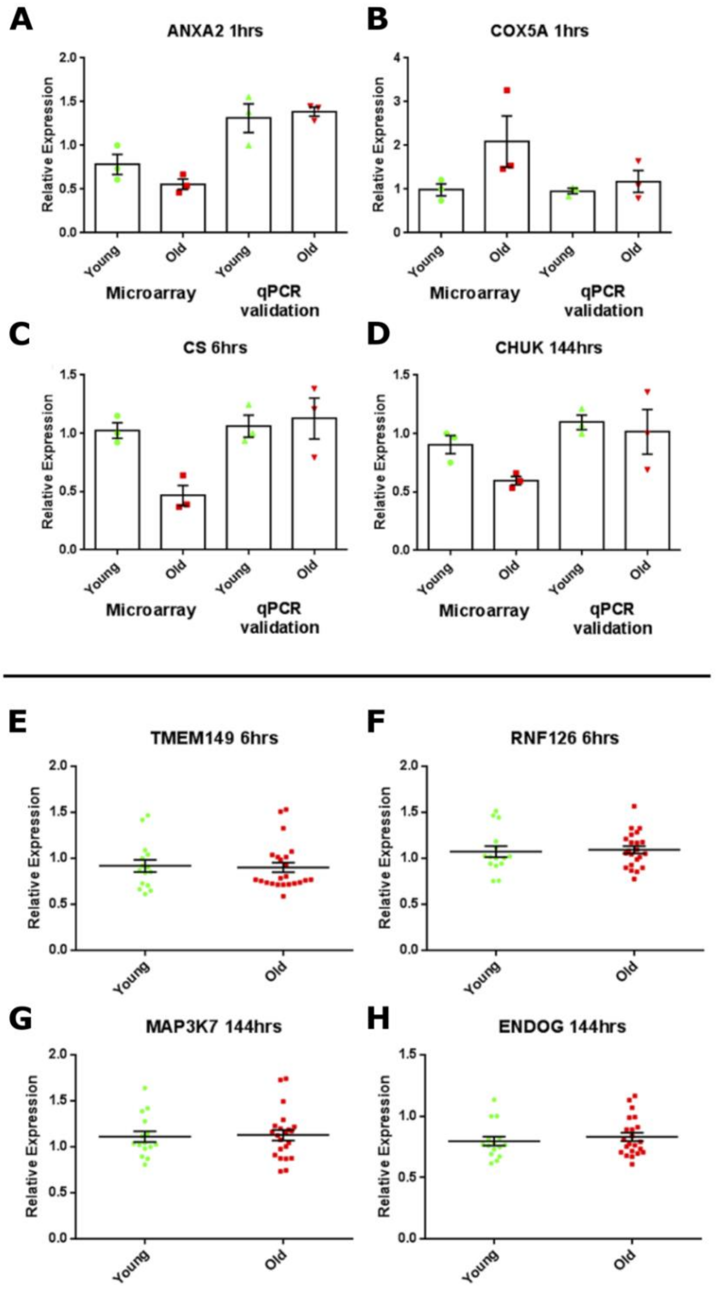

Supplementary Figure 6. (A-D) Not all candidate genes differentially expressed by young or old serum in the microarray analysis are validated by qPCR: relative expression of microarray candidates **(A)** Annexin 2 (ANXA2) **(B)** cytochrome c oxidase subunit Va (COX5A) **(C)** citrate synthase (CS) **(D)** conserved helix-loop-helix ubiquitous kinase (CHUK) normalised to one young-serum induced readout, corresponding to 1 on the y axis for both the microarray and qPCR validation. Each green circle (young, n = 3) or red square (old, n = 3) represents expression values during differentiation of hippocampal progenitors in the presence of human serum at the stated time point. Microarray statistics not included. Unpaired student one-tailed t-test conducted on qPCR data, error bars = SEM. **(E-H) Validation by qPCR of differentially expressed microarray candidates' genes in response to young or old serum:** relative expression of microarray candidates **(E)** transmembrane 149 (TMEM149) **(F)** ring finger protein 126 (RNF126) **(G)** mitogen-activated protein kinase 7 (MAP3K7) **(H)** endonuclease G (ENDOG) normalised to one young subject (21 years), corresponding to 1 on the y axis. Each green circle (young serum, n = 17, mean age of 25.6 years) or red square (old serum, n = 23, mean age of 78 years) represents n = 2 technical replicates following analysis of qPCR data, after 6 hours or 144 hours differentiation of hippocampal progenitors in presence of human serum. Unpaired student and Mann Whitney two-tailed t-tests as appropriate, error bars = SEM.

Supplementary Tables:

© 2021. De Lucia C et al. Published online at http://www.aginganddisease.org/EN/10.14336/AD.2021.0409

SUPPLEMENTARY DATA

Supplementary Table 1. Epidemiological factors and cellular data old cohort attached as a separate excel file as part of supplementary materials.

Sample ID	Age (yrs)	Gender	Education (yrs)	MMSE	CDR	CERAD	Deterioration	Depression	Waist (cm)	Systolic	Disastolic	Anti-hypertensive	Inflam	Statins
DCR00013	82.0	M	21.0	30.0	0.0	9.0	1.0	2.0	NA	153.0	85.0	1.0	1.0	1.0
DCR00045	74.0	M	13.0	29.0	0.0	11.0	1.0	2.0	100.5	128.0	101.0	1.0	1.0	1.0
DCR00051	74.0	F	9.0	30.0	0.0	14.0	1.0	0.0	86.0	130.0	72.0	NA	NA	NA
DCR00091	73.0	F	17.0	30.0	NA	13.0	1.0	1.0	88.0	148.0	76.0	0.0	0.0	0.0
DCR00095	84.0	F	11.0	30.0	0.0	11.0	1.0	NA	NA	NA	NA	1.0	0.0	0.0
DCR00096	82.0	F	14.0	29.0	0.0	11.0	1.0	NA	NA	NA	NA	NA	NA	NA
DCR00099	69.0	F	12.0	30.0	NA	12.0	1.0	1.0	75.0	128.0	73.0	0.0	0.0	1.0
DCR00164	79.0	M	17.0	30.0	0.0	NA	2.0	1.0	104.0	157.0	74.0	0.0	1.0	1.0
DCR00280	94.0	M	11.0	30.0	0.0	6.0	2.0	4.0	93.0	164.0	84.0	1.0	1.0	1.0
DCR00311	75.0	F	12.0	30.0	NA	13.0	1.0	1.0	74.0	174.0	82.0	NA	NA	NA
DCR00320	73.0	F	14.0	30.0	NA	11.0	1.0	3.0	NA	117.0	76.0	1.0	1.0	0.0
DCR00434	70.0	M	15.0	29.0	0.5	14.0	2.0	2.0	93.0	139.0	77.0	NA	NA	NA
DCR00464	73.0	M	10.0	28.0	0.0	7.0	1.0	1.0	82.0	152.0	90.0	0.0	1.0	0.0
DCR00507	77.0	F	11.0	30.0	0.5	8.0	2.0	3.0	NA	NA	NA	NA	NA	NA
DCR00512	91.0	F	NA	30.0	NA	7.0	2.0	0.0	95.0	169.0	89.0	1.0	0.0	1.0
DCR00517	78.0	F	14.0	29.0	0.0	8.0	1.0	1.0	NA	NA	NA	0.0	1.0	0.0
DCR00518	77.0	M	10.0	25.0	0.0	3.0	1.0	2.0	116.0	153.0	91.0	1.0	1.0	0.0
DCR00521	85.0	M	11.0	26.0	0.0	4.0	1.0	3.0	107.0	148.0	76.0	1.0	1.0	0.0
DCR00549	81.0	M	10.0	29.0	0.0	11.0	1.0	0.0	NA	NA	NA	1.0	0.0	1.0
DCR00727	69.0	M	16.0	29.0	NA	10.0	1.0	4.0	NA	NA	NA	0.0	0.0	0.0
DCR00802	71.0	F	16.0	27.0	0.0	9.0	1.0	0.0	96.0	154.0	89.0	1.0	1.0	0.0
DCR00803	72.0	M	28.0	30.0	0.0	14.0	1.0	0.0	86.0	128.0	73.0	1.0	0.0	0.0
DCR00805	73.0	M	15.0	30.0	0.0	6.0	2.0	0.0	90.0	149.0	93.0	1.0	0.0	0.0
DCR00806	80.0	F	6.0	27.0	0.5	8.0	1.0	5.0	91.0	133.0	69.0	0.0	1.0	0.0
DCR00808	80.0	F	10.0	29.0	NA	7.0	1.0	1.0	83.0	152.0	85.0	1.0	1.0	1.0
DCR00819	78.0	M	11.0	29.0	0.5	11.0	2.0	2.0	96.0	142.0	92.0	1.0	0.0	1.0
DCR00824	73.0	F	18.0	28.0	NA	10.0	2.0	5.0	NA	144.0	81.0	1.0	0.0	1.0
DCR00884	70.0	M	11.0	28.0	NA	11.0	1.0	1.0	102.0	165.0	96.0	1.0	0.0	1.0
DCR00892	74.0	F	10.0	28.0	NA	7.0	2.0	1.0	72.0	141.0	66.0	1.0	1.0	1.0
LNDCTL059	84.0	F	10.0	NA	NA	NA	NA	NA	NA	NA	NA	NA	NA	NA
LNDCTL061	76.0	F	16.0	30.0	0.0	NA	1.0	0.0	NA	NA	NA	0.0	1.0	0.0
LNDCTL071	78.0	F	12.0	29.0	0.0	NA	1.0	2.0	93.0	NA	NA	NA	NA	NA
LNDCTL076	93.0	F	11.0	28.0	0.5	NA	2.0	2.0	87.5	NA	NA	1.0	1.0	1.0
LNDCTL077	76.0	M	16.0	29.0	0.0	NA	1.0	3.0	NA	NA	NA	1.0	0.0	0.0
LNDCTL081	79.0	F	15.0	29.0	0.0	NA	1.0	1.0	74.0	NA	NA	0.0	1.0	0.0
**Average**	77.5		13.3	28.9		9.0		1.7	90.6	146.4	82.2	NA	NA	NA
**Std. deviation**	6.6		**3.9**	**1.3**		3.0		1.5	11.5	14.5	8.8			

© 2021. De Lucia C et al. Published online at http://www.aginganddisease.org/EN/10.14336/AD.2021.0409

SUPPLEMENTARY DATA

Supplementary Table 1 Cont.

Sample ID	Ki67 (%)	Cell Number, Av/field	CC3 (%)	Map2 (%)	Dcx (%)
DCR00013	17.6	718.3	16.4	15.6	11.0
DCR00045	20.1	706.7	4.9	NA	4.2
DCR00051	20.2	910.9	3.7	23.9	12.1
DCR00091	5.1	212.2	37.3	18.6	29.8
DCR00095	23.1	505.6	2.1	20.1	18.9
DCR00096	29.4	539.1	2.8	9.8	11.4
DCR00099	2.3	205.7	8.3	16.6	19.9
DCR00164	14.8	557.4	10.3	7.5	10.0
DCR00280	19.9	524.4	17.1	13.2	16.6
DCR00311	26.3	549.8	4.1	15.3	14.2
DCR00320	10.1	611.7	1.2	11.6	8.9
DCR00434	5.4	204.4	35.0	18.6	17.5
DCR00464	23.9	505.8	3.6	9.0	8.5
DCR00507	17.5	567.4	2.0	12.8	12.7
DCR00512	22.9	730.8	5.5	8.9	4.5
DCR00517	21.8	753.6	4.1	10.1	3.9
DCR00518	20.7	641.2	8.9	19.0	10.1
DCR00521	19.0	695.7	4.9	19.7	11.9
DCR00549	18.7	696.6	6.3	22.7	9.8
DCR00727	9.0	554.7	39.9	18.2	16.2
DCR00802	25.3	614.4	8.2	NA	3.2
DCR00803	27.3	540.9	16.2	NA	7.9
DCR00805	18.2	603.7	10.5	6.4	11.4
DCR00806	9.4	261.3	37.3	19.7	24.7
DCR00808	19.6	246.0	32.4	22.0	26.5
DCR00819	17.1	475.5	18.0	5.9	14.1
DCR00824	27.3	560.7	22.3	NA	13.2
DCR00884	18.0	558.8	15.9	7.3	14.5
DCR00892	20.1	474.0	15.8	7.0	8.5
LNDCTL059	29.1	571.1	3.0	11.6	10.9
LNDCTL061	8.1	475.8	5.2	5.2	4.2
LNDCTL071	17.4	806.4	3.5	16.5	8.8
LNDCTL076	17.7	564.0	3.0	12.6	7.7
LNDCTL077	14.3	752.7	2.1	8.9	6.2
LNDCTL081	14.8	610.7	2.3	19.4	13.1

Supplementary Table 1 Key

NA	Datapoint not measured during serum sample collection
NA	Datapoint removed owing to increased variation among technical replicates
MMSE	Mini-mental state examination
CDR	Clinical Dementia Rating (0 and 0.5 is cognitively normal)
CERAD	Consortium to Establish a Registry for Alzheimer's Disease scale (scores closer to 24 reflect more cognitive dysfunction)
Deterioration	Global Deterioration Scale (1 = no cognitive decline, 2 = very mild cognitive decline)
Depression	Geriatric Depression Scale (0-9 = normal affect)
Anti-hypertensive	1 = currently taking anti- hypertensive medication, 0 = no medication
Inflam	1 = presence of inflammatory disease e.g. arthritis, 0 = no disease
Statins	1 = currently taking statins, 0 = no statins. NA = information not available. SD = standard deviation about mean.

© 2021. Lucia CD et al. Published online at http://www.aginganddisease.org/EN/10.14336/AD.2021.0409

SUPPLEMENTARY DATA

Supplementary Table 2. Range of anti-hypertensives taken by old subjects.

Antihypertensive	Mechanism of action
**Nifedipine**	Calcium antagonist, vasodilator
**Ramipril**	Angiotensin-converting enzyme inhibitor
**Warfarin**	Anticoagulant
**Amlodipine**	Calcium channel blocker
**Aspirin**	Non-steroidal anti-inflammatory drugs
**Natrilix**	Modifies calcium exchange, vasodilating effect
**Lyprinol**	Omega 3 fatty acid, anti-inflammatory
**Bisoprolol**	β-blocker
**Isosorbide mononitrate**	Vasodilator
**Furosenide**	A loop diuretic, targets Na-KCL transporter
**Digoxin**	Inhibition of the Na+/K+ ATPase, mainly in the myocardium, steady heart rate
**Enalapril**	Angiotensin-converting enzyme inhibitor
**Doxazosin**	α1-selective alpha blocker
**Omemprazole**	Proton pump inhibitor, relieve heartburn
**Bendroflumethiazide**	Inhibit sodium absorption, vasodilator

© 2021. Lucia CD et al. Published online at http://www.aginganddisease.org/EN/10.14336/AD.2021.0409

SUPPLEMENTARY DATA

Supplementary Table 3. Full list of target molecular hallmarks of ageing assayed.

Gene		Function
**NAMPT**	Nicotinamide phosphoribosyltransferase	Catalyses a key intermediate step in the production of nicotinamide adenine dinucleotide (NAD), a key co-factor for enzymes involved in mitochondrial function and metabolism.
**DIABLO**	Diablo homologue	Mitochondrial protein released into the cytosol during apoptosis.
**DNML1**	Dynamin 1-like	Mediates mitochondrial division.
**SOD2**	Superoxide dismutase 2	Clears mitochondrial Reactive Oxygen Species and so protects against cell death. Upregulated in response to oxidative stress.
**POLG**	-	DNA polymerase subunit gamma (POLG) is a catalytic subunit of the mitochondrial enzyme DNA polymerase gamma needed for mitochondrial replication.
**PARP1**	Poly ADP-ribose polymerase 1	PARP1 activity is required for genome stability; acts via several different DNA repair pathways.
**XRCC2**	X-ray repair complementing defective repair in Chinese hamster Cells 2	Encodes a member of the RecA/Rad51-related protein that is required for the repair of double stranded DNA breaks by homologous recombination.
**TERT**	Telomerase reverse transcriptase	A catalytic subunit of the enzyme telomerase, a key enzyme which maintains the length of telomeres which otherwise shorten following mitosis and is essential for maintaining genome integrity.
**SIRT1**	Silent mating type information regulation 2 homolog 1	An enzyme that deacetylates proteins involved in cellular stress responses and longevity. Deacetylation is an epigenetic mechanism that regulates gene expression.
**UCHL1**	Ubiquitin carboxyl-terminal esterase L1	Neuron-specific enzyme involved in the ubiquitin-proteasome system.
**ATF4**	activating transcription factor 4	Required for autophagy.
**ATG5**	autophagy protein 5	Required for autophagy.
**pS6**	phosphorylated S6	Ribosomal protein kinase (S6) is a downstream component of the target of rapamycin complex 1 (TORC1) and functions as a key regulator of autophagy, protein synthesis and cell metabolism.
**AMPK**	AMP-activated protein kinase	A highly-conserved regulator of cellular energy homeostasis. This kinase is activated in response to stresses that deplete cellular ATP supplies such as low glucose, hypoxia, ischemia, and heat shock; activation results in both upregulation of catabolic processes to generate more ATP and inhibition of anabolic pathways.
**AKT**	Protein Kinase B	conserved kinase that regulates glucose metabolism and other key cellular processes such as growth-factor mediated proliferation and apoptosis
**CDKN2A/P16$^{\mathbf{Ink4a}}$**	Cyclin-dependent kinase inhibitor 2A	Encodes key proteins that regulate the cell cycle.
**CDKN1A/p21$^{\mathbf{Cip1/Waf1}}$**	cyclin-dependent kinase inhibitor 1A	Encodes key proteins that regulate the cell cycle.
**CDKN2B/p15$^{\mathbf{Ink\text{-}4b}}$**	cyclin-dependent kinase inhibitor 2B	Encodes key proteins that regulate the cell cycle.
**TP53**	tumour protein 53	Encodes key proteins that regulate the cell cycle.
**FADD**	Fas-associated protein with death domain	Adaptor protein that links TNFα signalling to apoptosis.
**TNFR1**	Tumour necrosis factor receptor 1	Binds TNFα and mediates both activation of the transcription factor nuclear factor kappa-light-chain- enhance of activated B cells (NF-κB) and apoptosis.
**NF-κB**	Nuclear factor kappa-light-chain-enhancer of activated B cells	Controls transcription, cytokine production and cell survival.

© 2021. Lucia CD et al. Published online at http://www.aginganddisease.org/EN/10.14336/AD.2021.0409

SUPPLEMENTARY DATA

Supplementary Table 4. Candidate list of genes returned by young versus old analysis of microarray data attached as a separate excel file as part of supplementary materials.

Rank	Gene Name	P Value	Rank	Gene Name	P Value
1	**ISCA1L**	0.000065	71	**SPATA7**	0.005144
2	**ANXA2**	0.000144	72	**SPC25**	0.005170
3	**HS.544736**	0.000157	73	**CHUK**	0.005194
4	**ATP6AP1**	0.000383	74	**LOC441155**	0.005257
5	**ARPP19**	0.000398	75	**LOC727935**	0.005390
6	**HS.564949**	0.000434	76	**SLC19A3**	0.005411
7	**PFTK1**	0.000499	77	**PPIL5**	0.005527
8	**LOC100130446**	0.000567	78	**EIF2S2**	0.005663
9	**C7ORF47**	0.000605	79	**CCDC97**	0.005665
10	**TUBB**	0.000643	80	**TDRD7**	0.005723
11	**LOC643319**	0.000724	81	**FAM50B**	0.005760
12	**LOC652161**	0.000823	82	**VHL**	0.005808
13	**HS.280461**	0.001002	83	**THUMPD2**	0.005892
14	**ASAP2**	0.001036	84	**TNFRSF12A**	0.005940
15	**PPAP2A**	0.001079	85	**LOC401233**	0.005966
16	**ABTB1**	0.001172	86	**LOC645605**	0.005977
17	**TMEM149**	0.001187	87	**CCDC12**	0.006085
18	**ZC4H2**	0.001199	88	**TTC12**	0.006128
19	**C3ORF72**	0.001283	89	**SCARNA27**	0.006142
20	**ZNF264**	0.001298	90	**HS.254006**	0.006238
21	**IFIT3**	0.001302	91	**POLM**	0.006310
22	**ZMYM2**	0.001335	92	**PYCR2**	0.006318
23	**ZDHHC6**	0.001368	93	**HS.539591**	0.006343
24	**HS.187499**	0.001457	94	**MTMR12**	0.006403
25	**HS.565209**	0.001604	95	**TRAF6**	0.006667
26	**ENDOG**	0.001670	96	**LOC728142**	0.006734
27	**OTUD4**	0.001697	97	**RBMS1**	0.006770
28	**PRDX3**	0.001814	98	**HLA-DPB1**	0.006819
29	**TRIM45**	0.001920	99	**DCTPP1**	0.006906
30	**VARS**	0.001989	100	**LOC732381**	0.006925
31	**LCN1L1**	0.002010	101	**LOC644096**	0.006980
32	**FOXI3**	0.002061	102	**BATF3**	0.007178
33	**WDR89**	0.002172	103	**HS6ST2**	0.007260
34	**C9ORF116**	0.002285	104	**PHF6**	0.007367
35	**KCTD12**	0.002302	105	**LOC729417**	0.007423
36	**ANKRD33**	0.002414	106	**LMF2**	0.007475
37	**GNS**	0.002435	107	**IARS**	0.007560
38	**PDCD10**	0.002544	108	**MIPEP**	0.007615
39	**KIAA1128**	0.002550	109	**MRPS24**	0.007669
40	**ABI2**	0.002580	110	**ARL3**	0.007739
41	**WDR7**	0.002591	111	**HS.47995**	0.007858
42	**NDUFA13**	0.002701	112	**EEF1E1**	0.007864
43	**ATP9B**	0.002701	113	**MGC35361**	0.007917
44	**COL4A1**	0.002757	114	**COLQ**	0.007950
45	**BRP44L**	0.002768	115	**GRN**	0.008218
46	**PSMB10**	0.002900	116	**MAP7D2**	0.008389
47	**CASC4**	0.003074	117	**LOC643287**	0.008420
48	**MAP3K7**	0.003170	118	**RB1**	0.008452
49	**SDCCAG3**	0.003212	119	**CALHM2**	0.008476

© 2021. Lucia CD et al. Published online at http://www.aginganddisease.org/EN/10.14336/AD.2021.0409

50	**HS.296031**	0.003567	120	**MRPL34**	0.008563
51	**CTSD**	0.003651	121	**TSTD2**	0.008704
52	**C22ORF36**	0.003754	122	**HS.581662**	0.008708
53	**HS.567759**	0.004087	123	**LOC100128476**	0.008762
54	**LOC148413**	0.004101	124	**FAM65A**	0.008805
55	**ASF1A**	0.004197	125	**AMD1**	0.008818
56	**RNF126**	0.004209	126	**LOC644745**	0.008871
57	**ZFP91**	0.004319	127	**KILLIN**	0.008877
58	**DDX19B**	0.004433	128	**USP12**	0.008972
59	**IP6K1**	0.004526	129	**LOC441054**	0.009006
60	**COX5A**	0.004551	130	**RPL23AP7**	0.009076
61	**C8ORF44**	0.004610	131	**PDE12**	0.009084
62	**SNX12**	0.004656	132	**TULP3**	0.009096
63	**MRTO4**	0.004785	133	**TUBA1C**	0.009235
64	**HSPA2**	0.004823	134	**CRTAP**	0.009265
65	**CS**	0.004851	135	**MLL5**	0.009288
66	**ATP5EP2**	0.004941	136	**SNF8**	0.009325
67	**CYB5R3**	0.004952	137	**SFRS3**	0.009338
68	**OR1L8**	0.005021	138	**PDE8B**	0.009347
69	**LRFN1**	0.005038	139	**LOC100129243**	0.009429
70	**SRRM2**	0.005066	140	**LOC727849**	0.009531
			141	**APBA3**	0.009651

P value refers to magnitude of differential expression in response to young or old serum at either the 1, 6, 24, 72 or 144 time point during differentiation. 141 genes with a P value < 0.01, uncorrected for multiple comparisons.

Supplementary Table 5. List of primary antibodies.

Antibody (Host species / company)	Dilution	Function
BrdU (Rat / OBT0030CX, Serotech)	1:500	Synthetic analog of thymidine, labels proliferating cells during DNA replication
Ki67 (Rabbit / Ab15580, Abcam)	1: 500	Present during all active phases of the cell cycle (G1, S, G2, and mitosis) but absent from resting cells (G0)
CC3 (Rabbit / 9664,Cell Signaling)	1: 500	Cleaved Caspase 3 is a critical executioner of apoptosis
Sox2 (Rabbit / Ab5603, Abcam)	1: 500	A transcription factor essential for maintaining self-renewal and maintenance of neural stem cells
Nestin (Mouse / Mab5326, EMD Millipore)	1:1000	Intermediate filament protein and widely employed marker of neural stem cells
H2A.X (Mouse / 05-636-I, EMD Millipore)	1:500	A variant histone required for checkpoint-mediated cell cycle arrest and DNA repair following double-stranded DNA breaks
Dcx (Rabbit / Ab11267, Abcam)	1:500	Doublecortin is a microtubule-associated protein expressed by neuronal precursor cells and immature neurons in embryonic and adult cortical structures.
Map2 (Mouse / Ab11267, Abcam)	1: 500	Microtubule associated protein 2 is involved in microtubule assembly. The products of similar genes in rat and mouse are neuron-specific cytoskeletal proteins and are enriched in dendrites.
S100β (Rabbit / Z0311, DAKO)	1:1000 - 10,000	S100 calcium-binding protein B is expressed primarily by astrocytes and acts as a neurotrophic factor and neuronal survival protein

© 2021. Lucia CD et al. Published online at http://www.aginganddisease.org/EN/10.14336/AD.2021.0409

SUPPLEMENTARY DATA

Supplementary Table 6. Primer sequences for target genes.

Target gene	Forward (5' to 3')	Reverse (5' to 3')
PARP-1	CTGGAGAGAGATTCTGTTGCATAG	GGAAACCAGTAAGGCAGACAT
SIRT-1	GCAGTGTGAGTAGAAGGAAGTC	TCCTGAAATTCTTAGCACCAAGT
NAMPT	CAGAGCTCCCAGACTGC	CGTTGCTTAAGTCACTGCTC
PUMA	GTGACCACTGGCATTCATTTG	TCCTCCCTCTTCCGAGATTT
DIABLO	CTGAGGCCAAGGAGTGAAA	CTGTTGATGTTAAGTCCTGTTGAG
TNFR1	CCGCCTACTTGGTGCTAAC	GTCCCTCATCCTCGCAAAC
FADD	TCTCCTCTCTGAGACTGCTAAG	AGAGAGTGCTGTGTGTCAATC
DNM1L	GCCATAGTCCTCCAAGAAGAAA	AGGACAGGTGGAATACAATGAC
SOD2	GGATTGATGTGTGGGAGCA	CTCCCAGTTGATTACATTCCAAATAG
POLG	CCTACAAGCTGGGTCTGAATG	AATCCATGGTCACTTCCTTCC
ATP5EP2	GGACTCAGCTACATCCGATACT	TTACGCTGTTGCCAGAAGTC
NDUFA13	CACGGCTTCATGTGGTACA	CCAGGTCTGCAGAGCATTTA
CDKN2A	GCACATTCATGTGGGCATTT	GACTCAAGAGAAGCCAGTAACC
CDKN1A	CCAGCCTCTGGCATTAGAATTA	CGGGATGAGGAGGCTTTAAATA
TP53	AGGGATGTTTGGGAGATGTAAG	CCTGGTTAGTACGGTGAAGTG
CDKN2D	GGGAGCGTCCCAAATCAATA	CGCTGTACAAGAGACTGGAAA
CDKN2B	GTGGGAGAAGGCAGTGATTAG	CTCCACTTTGTCCTCAGTCTTC
XRCC2	ACGCCTTCCTTATGCCATATAC	GCCTATGTCCTGAATGGTACTG
UCHL1	GGGAGGGACTTTGCTGATTT	CGTGTCTGCAGAACAGAAGAA
ATG5	CAACTGGGCTGGTCTTACTT	GTGGTCCGGTAAGTCTTTCAT
ATF4	GGAGATAGGAAGCCAGACTACA	GGCTCATACAGATGCCACTATC
TERT	ACCTGCCGTCTTCACTTC	GGGATGGACTATTCCTATGTGG
RANBP17	GGATCCTGGATTGAGACGAA	GTGCTTCCAGGCTCGTTCTA

Supplementary Table 7. Primer sequences for housekeeping genes.

Target gene	Forward (5' to 3')	Reverse (5' to 3')
VIM	ctttgccgttgaagctgcta	gaaggtgacgagccatttcc
RPLP2	cagaggagaagaaagatgagaagaa	ctttatttgcaggggagcag
ACTG1L	GGCTGAGTGTTCTGGGATTT	GGCCAAAGACATCAGCTAAGA

© 2021. Lucia CD et al. Published online at http://www.aginganddisease.org/EN/10.14336/AD.2021.0409

SUPPLEMENTARY DATA

Supplementary Table 8. Lysis buffer reagents.

RIPA reagent (Company)	Concentration	Inhibitors (company)	Concentration
Tris pH 7.2 (T3253, Sigma)	20mM	AEBSG (A1421.0100, VWR)	1mM
NaCl (424295000, Acros Organics)	150mM	Pepstatin A (A2205.0010, VWR)	1ug/ml
Triton X-100 (T9284, Sigma)	1.0%	Leupeptin (SC-215242, Santa Cruz)	10ug/ml
EDTA pH8 (T9284, E5134)	5mM	Aprotinin (14716, Cambridge Bioscience)	10ug/ml
Deoxycholate (D6750, Sigma)	1%	Ser/Thr phosphatase inhibitor Cocktail #3 (P0044, Sigma)	1:100
10% SDS (L4390, Sigma)	0.1%		
ddH$_2$0 (w4502, Sigma)	Top up to desired volume		

Supplementary Table 9. Primary antibodies used for detection of proteins.

Antibody (All cell signalling, UK)	Dilution	Function
**Akt (pan) (40D4) (Mouse / 2920BC)**	1:500	Part of conserved master age-regulating pathway; turned on by insulin, insulin-like hormone and insulin-like growth factors. Upon activation predominantly induces anabolic, synthetic and growth systems, and three of its targets mTOR, GSK3β (glycogen synthase kinase 3β) and FOXO ((Forkhead box-O class) transcription factors) are central to this.
**Phospho-Akt (Ser473) (D9E) XP® (Rabbit / 4060BC)**	1:500	Activated AKT – function as above
**AMPKAlpha (F6) (Mouse / 2793BC)**	1:500	AMP-activated protein kinase (AMPK) is a crucial cellular fuel sensor controlling fatty acid oxidation, mitochondrial biogenesis and insulin sensitivity. Reduced AMPK function is linked to ageing processes.
**Phospho-AMPKAlpha (Thr172) (40H9) (Rabbit / 2535BC)**	1:500	Activated AMPK – function as above
**NF-KappaB p65 (D14E12) XP® (Rabbit / 8242BC)**	1:500	The IKK/NF-κB signalling pathway has been proposed to be one of the key mediators of ageing; it is activated by genotoxic, oxidative, and inflammatory stresses and regulates expression of cytokines, growth factors, and genes that regulate apoptosis, cell cycle progression, cell senescence, and inflammation.
**Phospho-NF-KappaB p65 (Ser536) (7F1) (Mouse / 3036BC)**	1:500	Activated NF-KappaB p65 - function as above
**S6 Ribosomal Protein (54D2) (Mouse / 2317BC)**	1:500	Ribosomal S6 protein is a kinase and key component of the nutrient-responsive mTOR (mammalian target of rapamycin) signalling pathway that regulates cell growth and protein synthesis. Knockdown of this protein extends lifespan.
**Phospho-S6 Ribosomal Protein (Ser235/236) (Rabbit / 2211BC)**	1:500	Activated S6 – function above
**SirT1 (1F3) (Mouse / 8469BC)**	1:500	A key protein deacetylase, dependent on nicotinamide adenine dinucleotide (NAD) as a cofactor; upon activation helps extend lifespan across multiple species. Sirtuins deacetylate a wide range of histone and non-histone targets, and influence multiple aspects of cellular and organismal physiology and pathology
**Phospho-SirT1 (Ser27) (Rabbit / 2327BC)**	1:500	Activated SIRT1 – function above

© 2021. Lucia CD et al. Published online at http://www.aginganddisease.org/EN/10.14336/AD.2021.0409

SUPPLEMENTARY DATA

Supplementary Table 10. Primer sequences for target genes revealed by microarray study.

Target gene	Forward (5' to 3')	Reverse (5' to 3')
ANXA2	TACTTTGTGGCCCTGCTTTC	TCCCAGAGCTTTCTTCCTACA
CDK14	GCATGAAACCTAGCTCCTCTAC	GCACTCACTCATTCTCTCCTTC
IGFRL1	GCTGGAAGAGCTGATTGTACT	GCAGGCAGCCCATATCTT
ZNF264	GTCTGTGTCTTCCTTCCAACTC	CAGCAGGAACCATCTCATTCA
ENDOG	ACCAGAATGCCTGGAACAA	GCCCTGTGCAGACATAGAC
PDCD10	TGAATGAGGATGACAATGGAAGA	ACACAGGATACATGACTGCATAG
SDCCAG3	TGTTGATCGCAAGAGTCCAG	CCAAGAAGGGCTGCATAGAA
RNF126	CTCTGTCTAACCTCACCCTCTA	GCTCAAACGTCCGTTTATTTCA
ZFP91	GGCTGGGAGAGAGAGATTAGA	GCCACTGGAGAGCTATGATTT
COX5A	GCAGGACCTCATAAGGAAATCT	CCATGCGGTTTACACTTTGTC
CS	CTGGCACCCAATGTTTGATTT	GTCTGGGAGGAGGTGAGTATTA
WDR81	GAATCACCAGAGCCACCAA	CTTCCTCCTGCTCTTAACTTCC
OAT	ACCCATGGCGACATTATCAG	GGCTACCCTCAGAAAGACAAG
RFN135	GGGAAGTGGACACTAGGAATTG	CCACACAACAAGAGTCCATAGT
ATXN10	GTCTTGTCACTGAGCCCTATC	ACTTCTGGGATGAACTGGAAC
FBX011	caatggctggagtctggatt	ctcgacccccattaaatataca
SEM4A	GTCAGCCTTGGCCTCTTATT	CCAACTCCATCCTGCACTATC

© 2021. Lucia CD et al. Published online at http://www.aginganddisease.org/EN/10.14336/AD.2021.0409

SUPPLEMENTARY DATA

Supplementary Methods

ReNeuron's HPC0A07/03C cells with *c*-mycER^{TAM} technology

Owing to the genetically engineered *c*-mycER^{TAM} technology, in the presence of growth factors (4-OHT, FGF2 and EGF), HPC0A07/03C cells will proliferate indefinitely. Removal of these factors induces differentiation into hippocampal neurons, astrocytes and oligodendrocytes, but not into other cell types outside of the brain. For further information pertaining to both the generation of the genetic construct and the obtainment of foetal-brain tissue, please see Pollock et al., 2006.

Automated quantification of immunofluorescence

An automated approach was employed to quantifying cell number and markers of proliferation, differentiation and cell death using the CellInsight* NXT High Content Screening (HCS) Platform (ThermoScientific).

The development of specific paradigms for our assays and cellular markers was based on two BioApplications available within the Cell Insight machine software package: (1) Target Activation enabled quantification of nuclear markers (BrdU, Ki67, Sox2 and H2A.X) and (2) Cell Health Profiling enabled quantification of markers expressed in the cell body and dendrites quantify (Nestin, CC3, S100B, DCX and Map2). Target activation facilitates the assessment of co-labelling within the region of the nucleus, as such, only staining confined within nuclear perimeter as stipulated by DAPI staining is quantified. In contrast, cell health profiling relies upon nuclear staining to aid construction of a concentric circle around the immediate vicinity of the nucleus (i.e., around most of the cell body). The creation of such a circle creates a map or region of interest. Staining confined within the region between the outer circle (edge of cell body) and inner circle (outside of the nucleus) is deemed positive.

Total RNA extraction and quantification of concentration and purity

Cells seeded at a density of $3x10^5$ in 6-well plates (Nunclon, Denmark) and treated with young and old human serum were harvested and pelleted at 6 hour and 6-day time points during the differentiation assay (Supp. Figure 7). First, supernatant was completely aspirated, and the cells were immediately lysed by scraping and resuspension in 1ml of TRIreagent® (T9424, Sigma) and stored at -80^{oC} until further use.

2ml sized Phase Lock Gel Heavy (2302830, 5 Prime) tubes were pre-spun at 13000rpm for 2 minutes and entire contents of thawed samples (1ml of re-suspended cells in TRIreagent) were transferred into these tubes. Chloroform is then added at a 5:1 ratio, in this case 200µl, to the tubes and mixed by vigorous shaking for 5-10 seconds. The mix is left at room temperature for 3 minutes where the layers start to separate out with a translucent layer containing RNA on the top. After centrifugation at 10,000 rpm for 5 minutes at 4°C, the aqueous top layer is collected into a new Eppendorf tube, 500 µl of isopropanol (2:1 ratio with chloroform) is added and mixed by inversion x10 times. This is left to stand at room temperature for 15 minutes whilst isopropanol precipitates out nucleic acids, 80-85% of precipitate contains RNA. After 15 minutes, samples are centrifuged at 13,000 rpm for 15 minutes at 4°C. The small glass-like looking pellet is carefully washed in 80% ethanol and spun down at 14,000 rpm for 5 minutes at 4°C before air drying of pellet (10-15 minutes). Pellet re-suspended by gentle flick mix in molecular grade H_20 (certified RNAse free) for 15 minutes before adding 10µl of Sodium Acetate (R1181, Thermo Scientific) and 300µl of 100% ethanol, and is left at -80°C for 30 minutes or overnight. When ready for use, samples were spun down at 13,000 rpm for 15 minutes at 4°C and wash pellet in 80% ethanol before complete air-drying pellet (15-20 minutes). Re-suspend in 22.5µl of H_20, flick mix and leave on ice for 15 minutes before assessment of concentration and purity.

The concentration and purity of extracted nucleic acids was measured using the NanoDrop™ 1000 spectrophotometer (Thermo Scientific). The 260/280 and 260/230 absorbance ratios were used to assess the purity of RNA. A 260/280 ratio of >2.0 was considered pure for RNA and a 260/230 ratio of >2.0 was considered pure for RNA. To remove residual genomic DNA contamination, all RNA samples were treated with TURBO DNA-*free*™ (AM1907, Life Technologies).

© 2021. Lucia CD et al. Published online at http://www.aginganddisease.org/EN/10.14336/AD.2021.0409

SUPPLEMENTARY DATA

Reverse transcription

Complimentary DNA (cDNA) was synthesised using SuperScript® III Reverse Transcriptase (18080-044, Life Technologies). 1 µg of DNase-treated RNA was combined with 250 ng of random hexamers (N8080127, Life Technologies) and 1 mM dNTP mix (R0191, Thermo Scientific) made up to 13 µl with nuclease-free H20 (Sigma-Aldrich). The mix was incubated for 5 minutes at 65°C on a heated block to denature RNA secondary structure. It was then promptly placed on ice and incubated for 1 minute. The mix was then made up to 20 µl with the following reagents: 1x First Strand Buffer (Invitrogen), 5 mM Dithithretiol (18080-044, Life Technologies), 40 units RNaseOUT™ (10777, Life Technologies), 200 units SuperScript® III Reverse Transcriptase (Invitrogen) and 3 µl of nuclease-free H_20 (Sigma-Aldrich). Samples were incubated at 25ºC for 5 minutes, 50ºC for 1 hour (optimal temperature of Superscript® III), 55ºC for 30 minutes (to remove any secondary structures) and finally 70 ºC for 15 minutes to terminate the reaction. For qPCR applications, samples were diluted to a concentration of 1:30 and 1:40 in nuclease-free H_20.

Amplification primer design and testing of PCR specificity

PCR primer pairs were designed to amplify an amplicon of approximately 100 base pairs. DNA sequences for genes of interest were obtained from the University of California, Santa Cruz, genome browser (http://genome.ucsc.edu) and primers designed using integrated DNA technology (IDT) software (http://www.idtdna.com/primerquest/home/index) (Supp. Table 6). They were designed to have minimal self-complementarity and no complementarity to the other primer and were tested for single-peak melt curves using uMelt software (https://www.dna.utah.edu/umelt/umelt.html). The nucleotide sequence was sent to IDT who synthesised the oligonucleotides. They were re-suspended in nuclease-free H_20 to a stock concentration of 100 µM and used at a working concentration of 2 µM.

Lysate collection

Supernatant was aspirated from cells in a six-well plate before washing in ice-cold x1 PBS. PBS was completely removed to not dilute protein yield and cells were lysed on ice in 100µl of freshly made radioimmunoprecipitation assay (RIPA) buffer supplemented with several protease and phosphatase inhibitors (Supp. Table 8). Cells were scraped rigorously, and the lysate was collected into fresh Eppendorf tubes which were then sonicated (x5 pulses) and left on ice for 15-30 minutes. Lysates were spun down at 15,000 rpm for 15 minutes at 4ºC and the supernatant was collected into fresh tubes in preparation for protein concentration determination.

Determining protein concentration

Protein concentration was determined using the BCA assay (23225, Pierce BioTechnology, USA) as per manufacturer's instructions.

Preparing and loading samples

Samples were thawed on ice and denatured by mixing with laemmli X2 buffer (S3401, Sigma) at a 1:1 ratio and boiling at 95ºC for 5 mins on heat block. 12-well Mini Protean TGX 4-20% precast gels (456109DC, Biorad) were assembled into a green and white western unit filled it up with cold X1 SDS Electrophoresis (Running) Buffer diluted in ddH_20 from a X5 stock (15.1g Tris Base, 72g Glycine, 5g SDS, pH 8.4) and topped up with ddH_20 to 1L. Green combs were carefully removed, and each well was washed with x1 running buffer using a syringe to remove any air bubbles. 10µl of the kaleidoscope ladder (161-0375, Biorad) and 25ug of sample were loaded into appropriate wells.

Running gels and transferring to nitrocellulose membrane

The set-up involved first connecting the green and white plastic gel unit to an electrophoresis tank and filling this up with X1 running buffer until halfway up the tank. Gels were run at 100-120 V for 2 hours. Nitrocellulose membranes (77010, Life Technologies) were washed in X1 Western Transfer Buffer (WTB) containing 10% methanol diluted in ddH_20 from a 10x stock (30.3g Trizma base, 151.6g Glycine, pH 8.5 and topped up with ddH_20 to 1L). Membranes were further washed in ddH_20 and gels were dislodged from their plate and left to equilibrate in X1 WTB. Transfer

© 2021. Lucia CD et al. Published online at http://www.aginganddisease.org/EN/10.14336/AD.2021.0409
